# Radiological review of skull lesions

**DOI:** 10.1007/s13244-018-0643-0

**Published:** 2018-09-19

**Authors:** Carrie K. Gomez, Scott R. Schiffman, Alok A. Bhatt

**Affiliations:** Department of Imaging Sciences, University of Rochester, 601 Elmwood Avenue, Rochester, NY 14602 USA

**Keywords:** Skull, Calvarial, Benign, Malignant, Lesions

## Abstract

**Abstract:**

Calvarial lesions are often asymptomatic and are usually discovered incidentally during computed tomography or magnetic resonance imaging of the brain. Calvarial lesions can be benign or malignant. Although the majority of skull lesions are benign, it is important to be familiar with their imaging characteristics and to recognise those with malignant features where more aggressive management is needed. Clinical information such as the age of the patient, as well as the patient’s history is fundamental in making the correct diagnosis. In this article, we will review the imaging features of both common and uncommon calvarial lesions, as well as mimics of these lesions found in clinical practice.

**Teaching Points:**

*• Skull lesions are usually discovered incidentally; they can be benign or malignant.*

*• Metastases are the most frequent cause of skull lesions.*

*• Metastatic lesions are most commonly due to breast cancer in adults and neuroblastoma in children.*

*• Multiple myeloma presents as the classic “punched out” lytic lesions on radiographs.*

*• Eosinophilic granuloma is an osteolytic lesion with bevelled edges.*

## Introduction

Calvarial lesions are often asymptomatic and are usually discovered incidentally during computed tomography (CT) or magnetic resonance imaging (MRI) of the brain or as part of workup of local clinical symptoms or staging of other diseases [1–6]. Occasionally, they may present as a visible, palpable or symptomatic lump [1, 2, 4]. Clinical parameters such as the age and clinical history are important factors to guide the radiological diagnosis. Calvarial lesions may be benign or malignant; fortunately, benign tumours are the most commonly encountered lesions [1–6].

**Fig. 1:**
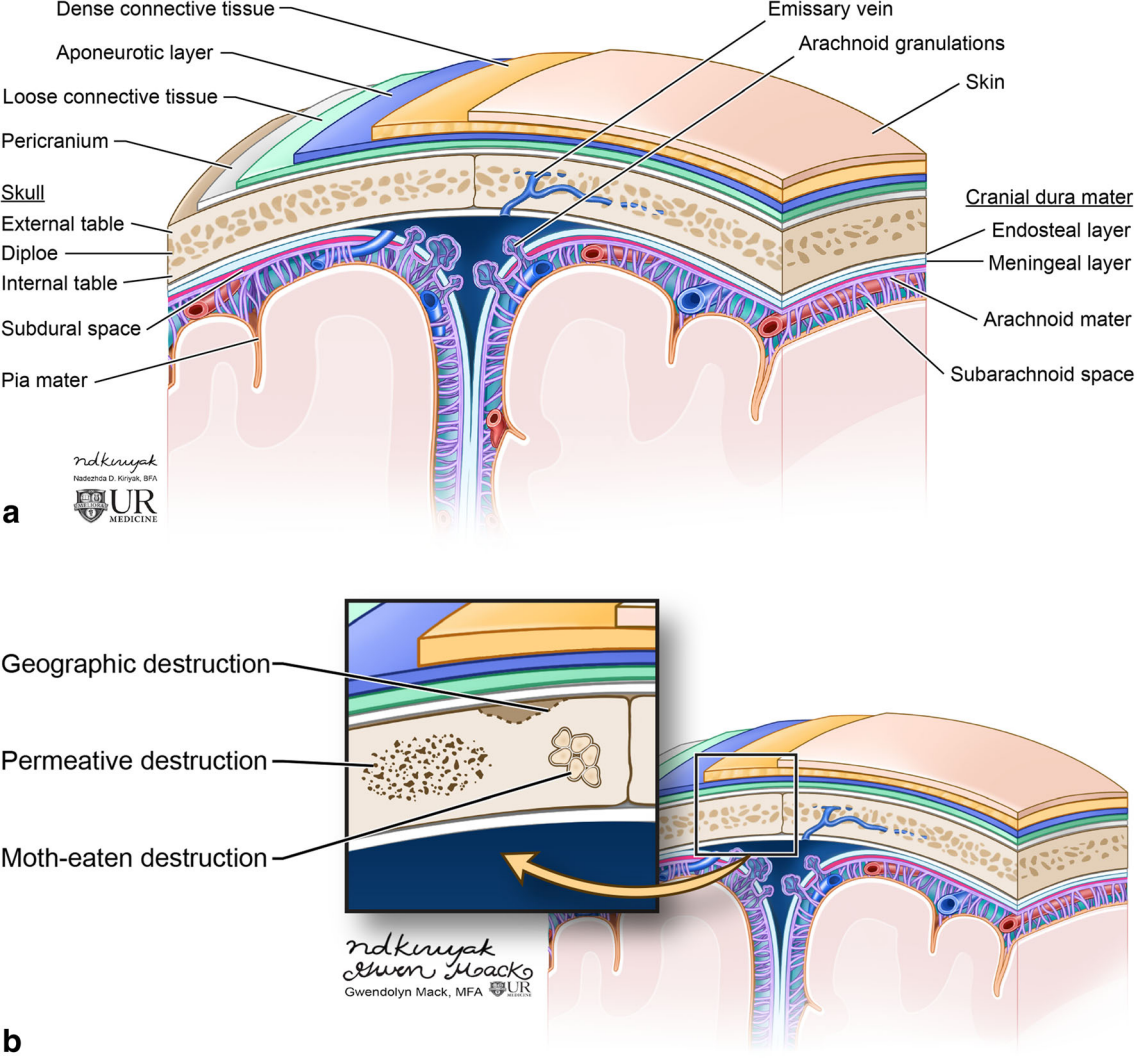
Image depicting the calvarium anatomy (**a**). The skull is composed of the marrow space (diploe), inner and outer tables. Covering the skull is the scalp which consists of the skin, subcutaneous dense connective tissue, galea aponeurotica and loose connective tissue. The outer table is covered by periosteum. Underneath the calvarium are the meninges comprised of the dura mater, arachnoid mater and pia mater. Diagram of the skull depicts patterns of bone destruction in the skull (**b**)

The skull vault is formed by the frontal, parietal, temporal and occipital bones and parts of the zygoma and sphenoid bone. It is composed of two cortical tables; the inner and outer tables, and the diploe or marrow space between them (Fig. 1) [1, 4]. Lesions of the calvarium may originate from the bony structures or may be secondary to invasion of scalp-based lesions or brain-based lesions into the skull vault [1, 4]. The skull base forms the floor of the cranial cavity and, therefore, similar lesions can occur in this region; however, there are lesions that are also specific to this location such as chordoma and chondrosarcoma.

Recognition of benign and malignant imaging features is important for the radiological diagnosis [1–6]. In general, benign tumours have well-defined borders with a narrow transition zone; sclerotic margins are frequently present. On the other hand, malignant tumours have poorly defined margins, a wide transition zone, aggressive periosteal reaction and often have a soft tissue component; these lesions cause dramatic bony destruction with intracranial or extracranial extension (Table 1) [1, 4]. Skull lesions can be lytic or sclerotic, single or multiple with varied composition; they may arise from osteogenic, chondrogenic, fibrogenic, vascular and/or other elements of bone (Tables 2 and 3).

**Table 1:** Features of benign and malignant lesions

Benign	Malignant
Geographic/sharp margins	Moth eaten or permeative bone destruction
Narrow zone of transition	Wide zone of transition
Sclerotic margin	Poorly defined margins
Periosteal reaction: solid/uninterrupted	Periosteal reaction: aggressive, interrupted
No soft tissue component	Soft tissue component

**Table 2:** Salient features of benign skull lesions on CT and MRI

Skull lesion	CT	MRI
Fibrous dysplasia	Typically homogeneously sclerotic with “ground-glass appearance”.	Variable signal depending on amount of mineralised stroma and fibrous tissue. Most commonly hypointense on T1 and T2.
Osteoma	Juxta-cortical sclerotic lesion.	Hypointense T1, variable signal on T2 depending on amount of cortical and trabecular bone.
Langerhans cell histiocytosis	Lytic lesion with “bevelled edges”. “Button sequestrum” may be present.	Variable signal, extensive marrow oedema is typically present. Plus enhancement.
Osseous venous vascular malformation (formerly haemangioma)	Trabeculations, “sunburst pattern”.	“Bunch of grapes” appearance. Diffuse enhancement.
Intraosseous meningioma	Sclerotic lesion. Hyperostosis.	Hypointense T1, variable signal on T2.
Paget disease	Lytic phase: “osteoporosis circumscripta”. Mixed phase (lytic and sclerotic): skull vault enlargement; “cotton-wool” appearance. Blastic phase: bone thickening and sclerosis.	Lytic phase: hyperintense T2, hypointense T1 (with foci of interspersed T1 hyperintense yellow marrow). Mixed phase: yellow marrow maintained in all sequences. Blastic phase: Hypointense T1 and T2.
Calvarial sarcoidosis (usually multiple)	Lytic. Well-demarcated margins.	Variable signal, may enhance. Can have periosseous soft tissue component.
Ossifying fibroma	Expansile, lytic lesion or solid lesion with areas of cystic changes.	Solid component is isointense on T1 and iso/hypointense on T2. Heterogeneous enhancement of the solid component.
Epidermoid cyst	Well-demarcated osteolytic lesion with sclerotic margins. Remodelling and expansion of skull tables.	Restricted Diffusion. Do not enhance.
Dermoid cyst (typically midline near anterior fontanelle)	Expansile osteolytic lesion. Can have soft tissue component.	Fatty signal on T1, variable signal on T2. May see peripheral enhancement.

**Table 3:** Salient features of malignant skull lesions on CT and MRI

Skull Lesion	CT	MRI
Multiple myeloma (multiple)	Osteolytic lesions without sclerotic rim, “punched-out lesions”.	Hypointense T1, hyperintense T2. “Salt and pepper marrow infiltration” is the most common pattern. Typically enhance.
Osteosarcoma	Lytic lesion, ill defined borders. Aggressive periosteal reaction. Variable amount of osteold matrix.	Variable appearance.
Metastases (may be solitary or multiple)	Lytic, sclerotic or mixed depending on the primary tumour.	Hypo/isointense T1, hyperintense T2. Typically enhance (unless sclerotic).
Chordoma (skull base, midline)	Lytic destructive expansile lesion of the clivus.	Hypointense T1, lobules of high signal on T2. Heterogeneous enhancement.
Chondrosarcoma (skull base, paramidline)	Osteolvtic lesion. Chondroid matrix with “rings and arcs”.	Hypo/isointense T1, hyperintense T2. “Whorls of enhancement”.

Calvarial lesions are radiologically evaluated with CT and MRI. CT is the most accurate method for evaluating bone destruction of the inner and outer tables, the lytic or sclerotic nature of the lesion and for the evaluation of mineralised tumour matrix [1–3, 6]. MRI is best to depict marrow involvement of the diploe and to evaluate the associated soft tissue component and invasion of adjacent tissues [1–3, 6]. Plain radiographs play a lesser role, but are useful in the assessment and follow-up of lytic lesions such as multiple myeloma; it may also be the initial modality on which a lesion is found [2, 6].

The differential diagnosis of calvarial lesions is important to decide whether biopsy, surgical intervention or conservative treatment is required for further management [1, 2, 6]. In this article, we will review the imaging characteristics of benign and malignant skull lesions, as well as systemic conditions affecting the skull. In addition, normal variants which may be mistaken for pathology, sometimes called “pseudolesions”, will also be reviewed.

## Benign calvarial lesions

### Fibrous dysplasia

Fibrous dysplasia represents 2.5% of all osseous and 7% of all benign osseous neoplasms [7]. Fibrous dysplasia results from abnormal differentiation and maturation of osteoblasts with progressive replacement of the normal bone by immature woven bone [1, 2, 5, 8, 9]. It is most commonly seen in adolescents and young adults and can be monostotic (70% of cases) or polyostotic (30% of cases) with the skull being involved in both forms of disease [1, 2, 4, 8, 9]. The monostotic form is the mildest and most common form; it involves the ribs and craniofacial bones, and is typically diagnosed between 20 and 30 years of age [1, 2, 8, 9]. The polyostotic form has an earlier onset, typically in childhood and affected patients tend to have more severe skeletal and craniofacial involvement; it may also be associated with McCune-Albright and Mazabraud syndromes [1, 2, 9]. Fibrous dysplasia preferentially affects the frontal and temporal bones and may cross sutures [2]. The diagnosis is often incidental, but may present as an enlarging mass with symptoms resulting from mass effect and narrowing of a foramen transmitting one of the cranial nerves [1–4, 8, 9]. Malignant transformation is very rare and has been reported in 0.4–1% of cases [2]. An associated aneurysmal bone cyst or pathological fracture may complicate this entity [2].

**Fig. 2:**
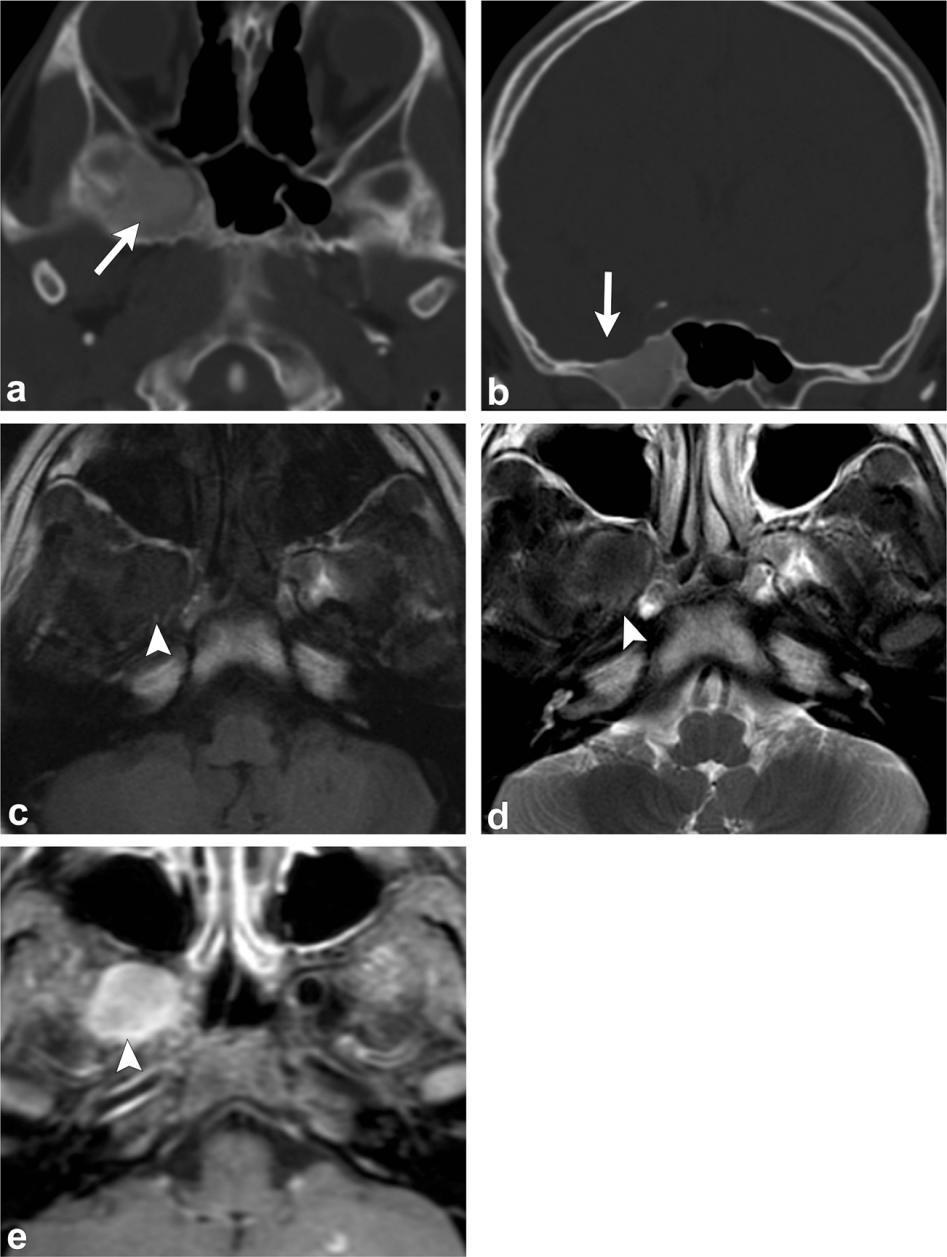
Fibrous dysplasia. Axial (**a**) and coronal (**b**) head CT images demonstrate an expansile lesion with ground-glass matrix in the right greater wing of the sphenoid (*arrows*). Axial T1-weighted (**c**), axial T2-weighted (**d**) and axial post-contrast T1-weighted (**e**) images show that the lesion is hypointense with homogeneous enhancement (*arrowheads*)

CT demonstrates an intradiploic, expansile lesion with the characteristic “ground-glass matrix” in all or part of the lesion (Fig. 2) [1–9]. The outer table is more prominently affected compared to the inner table [1, 2, 4]. Three patterns have been described: mixed lytic and sclerotic, homogeneously sclerotic and a predominantly cystic or lytic pattern; but the homogeneously sclerotic (ground-glass density) pattern is usually seen [1, 2, 4, 6]. Fibrous dysplasia can have varying signal and contrast enhancement on MRI depending on the ratio of fibrous tissue to mineralised matrix [1–9]. The lesion is typically low signal on T1- and T2-weighted images given that the homogeneously sclerotic form is most common (Fig. 2) [1–9]. Lesions with highly mineralised stroma tend to show lower signal intensities on T1- and T2-weighted images, whereas lesions with high fibrous tissue tend to have intermediate signal intensity on T1-weighted images and high signal on T2-weighted images [1, 2, 6, 8, 9]. It can be useful to get a CT if MRI findings are equivocal.

Treatment is based on bisphosphonates. Surgical decompression is performed when there is severe mass effect [2].

### Osteoma

Osteoma is the most frequent benign tumour in adults and is most commonly seen in men between the fourth and fifth decades of life [1, 2]. It is a juxta-cortical tumour made up of well-differentiated compact or cancellous bone [1, 2, 4–6]. Osteomas usually arise from the outer table and rarely from the inner table and can be sessile or pedunculated [1, 2, 4–6]. Inner table osteomas can be misdiagnosed as ossified meningiomas; however, unlike meningioma, osteomas do not have a soft tissue component and do not enhance [4, 6].

**Fig. 3:**
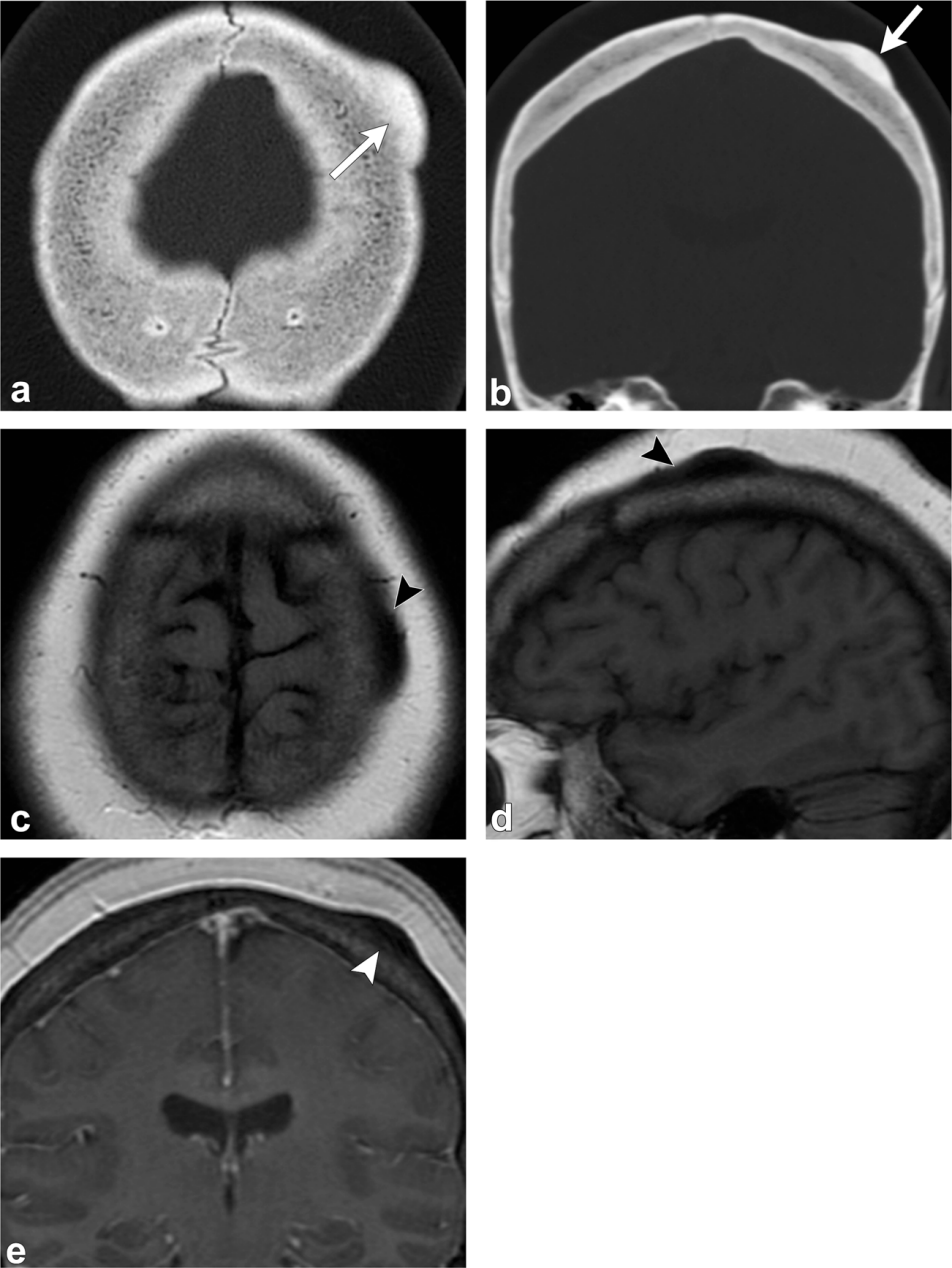
Osteoma. Axial (**a**) and coronal (**b**) head CT images show a juxta-cortical lesion along the outer table of the left frontal bone (*arrows*). Axial T1-weighted (**c**), sagittal T1-weighted (**d**) and coronal post-contrast T1-weighted (**e**) images depict the sclerotic/osteoblastic nature of this lesion (*arrowheads*). Note the well-defined margins and lack of enhancement

**Fig. 4:**
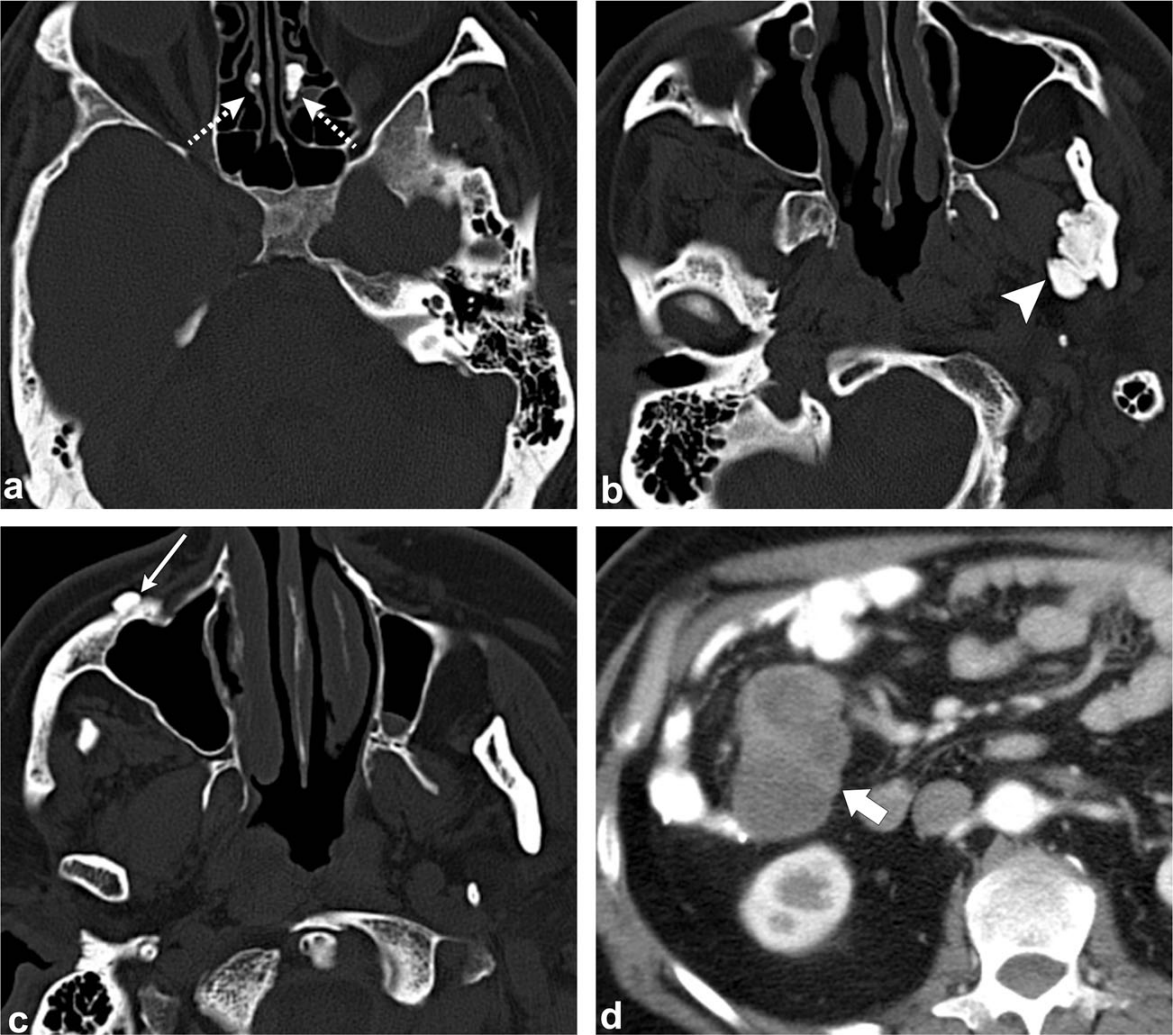
Gardner Syndrome (multiple osteomas). Axial head CT images (**a**–**c**) show multiple osteomas in the ethmoid air cells (*dashed arrows*), left ramus of the mandible (*arrowhead*) and right anterior wall of the maxillary sinus (*thin arrow*). This patient was also found to have a mesenteric desmoid tumour (*thick arrow*, **d**)

On CT, an osteoma is a juxta-cortical, well-defined, sclerotic, homogenous lesion [1, 2, 4–6]. On MRI, it has homogenous low signal on T1-weighted images, but variable appearance on T2-weighted imaging, depending on the amount of compact and cancellous/trabecular bone [1, 2, 4–6] (Fig. 3). When multiple osteomas are seen in the skull, Gardner syndrome should be considered, a disorder characterised by multiple osteomas, colonic polyposis, sebaceous cysts and various benign tumours such as lipomas and fibromas [1, 2] (Fig. 4).

Osteomas do not require treatment unless they cause mass effect and disturb surrounding structures [1, 2].

### Langerhans cell histiocytosis

Langerhans cell histiocytosis (LCH), formerly known as “histiocytosis X” is due to an idiopathic proliferation of Langerhans cells in various tissues (bone marrow, central nervous system, lung, liver, spleen, lymph nodes) causing focal or systemic disease [1, 2, 10, 11]. This disease is most common in children between 6 and 10 years of age and can occasionally be seen in young adults [1, 2, 4, 6, 10]. Langerhans cell histiocytosis comprises three clinical syndromes: (1) eosinophilic granuloma, which is limited to bone or lung; (2) Hand-Schuller-Christian disease, which presents with calvarial lesions, exophthalmos and diabetes insipidus; (3) Letterer-Siwe disease, the most aggressive form of LCH with multi-visceral organ involvement [1, 2, 10, 11]. The most frequent form of LCH is eosinophilic granuloma in which the calvarium is frequently involved [1, 2, 6]. Lesions more frequently occur in the parietal or frontal regions [2, 6]. Clinically, the lesions can be asymptomatic or present as palpable and/or tender masses [1, 2, 4, 6, 10].

On radiographs, there may be one or multiple well-defined punched-out osteolytic lesions [1, 2, 4, 6].

**Fig. 5:**
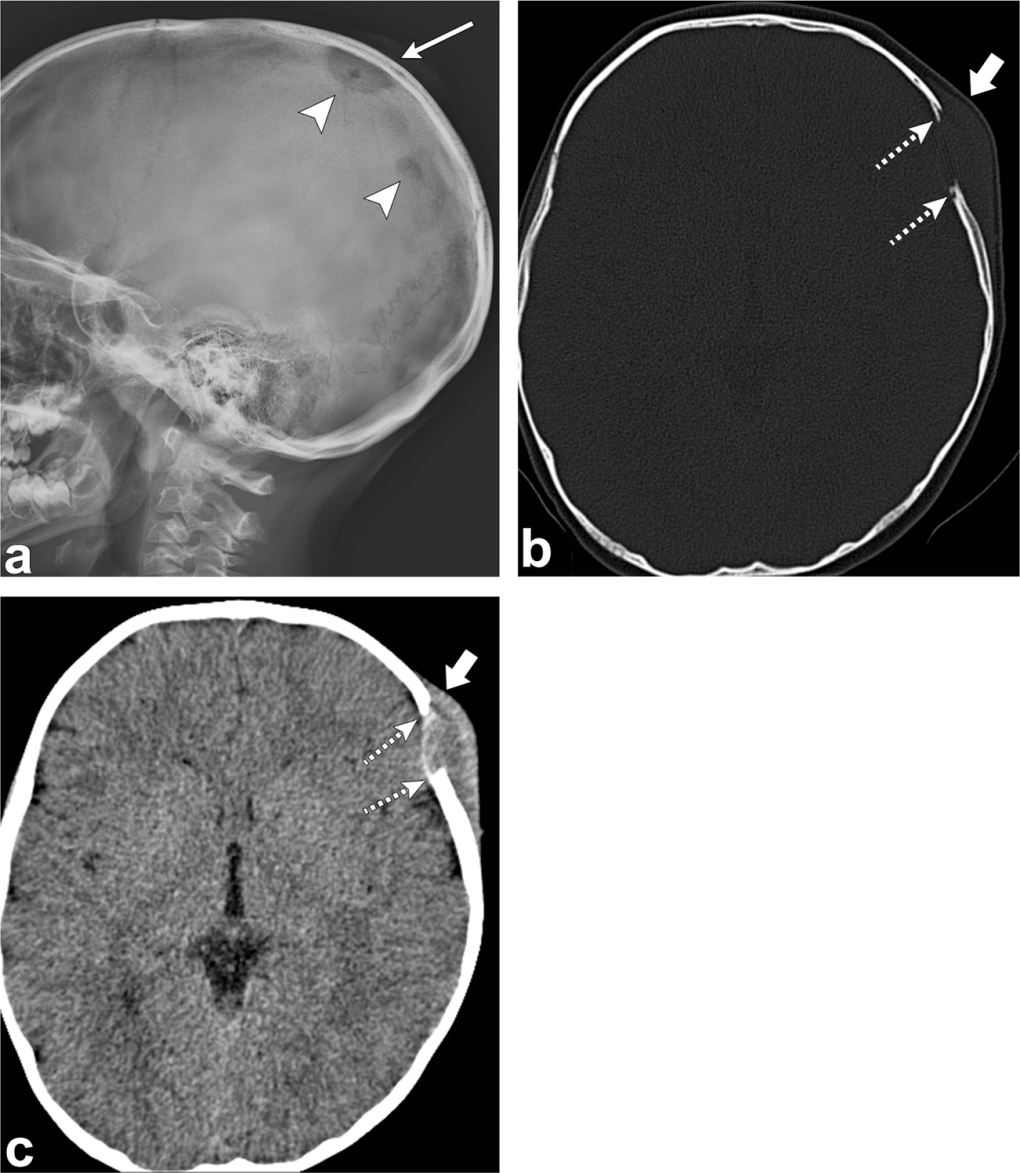
Langerhans cell histiocytosis. Sagittal skull radiograph (**a**) shows two lytic lesions (*arrowheads*) in the parietal skull, the largest one with an associated soft tissue component (*thin arrow*). Axial head CT images (**b**, **c**) in the bone and soft tissue windows in the same patient demonstrate an osseous destructive lesion in the left frontal calvarium with bevelled edges (*dashed arrows*) and a soft tissue component (*thick arrows*); pathology proven eosinophilic granuloma in a 4-year-old boy

On CT, the early appearance of LCH is an osteolytic lesion with bevelled edges due to unequal involvement of the inner and outer tables (Fig. 5) [1, 2, 4–6, 10]. The centre of the lesion may contain a “button sequestrum”, a central residual intact bone [1, 2, 6, 10]. They lack a sclerotic rim or periosteal reaction [1, 2]. Multiple lytic lesions may coalesce giving the appearance of a geographic map [2]. They can also have a soft tissue component and may have extradural and extracranial extension [1, 2, 5, 6]. Later on, healing lesions, whether spontaneous or under treatment, become sclerotic with centripetal bone formation and disappearance of the osteolytic lesions [2, 6].

MRI may aid in the depiction of bone marrow or soft tissue involvement, but the signal intensity of these lesions is non-specific; however, extensive bone marrow oedema is typically present [10, 12]. Most frequently, LCH has low to intermediate signal on T1-weighted images and high signal on T2-weighted images, except in the healing phase when the signal is low on T2 imaging [6]. Eosinophilic granuloma enhances strongly with gadolinium and often has reactive dural or galeal enhancement [1, 2, 5, 6].

Treatment varies depending on the severity and bony extent. Single lesions can be treated with surveillance or systemic corticosteroids. In the more aggressive forms of disease, surgical excision, radiotherapy and chemotherapy are treatment options [2].

### Osseous venous vascular malformation

The International Society for the Study of Vascular Anomalies (ISSVA) classification system divides vascular anomalies into two primary biological categories: vascular neoplasms and vascular malformations [13–15]. Vascular malformations include low-flow malformations (capillary, venous, and lymphatic), high-flow malformations (arterial malformation, arteriovenous malformation and arteriovenous fistula) and combined malformations (i.e. venolymphatic malformation). The previously called cavernous haemangiomas are actually venous malformations [13–15]. Osseous venous malformations are benign slow-growing vascular bone tumours that account for 2–10% of benign calvarial lesions and 0.2% of all bone neoplasms [1, 2, 16–18]. They affect the frontal and parietal bones predominantly and are more common during the 4th and 5th decades of life [1, 2, 6, 16–18]. Fifteen percent of skull venous malformations are multiple [18]. These lesions are more frequent in women than men with a ratio of 3:2 [2, 6, 16–18]. Calvarial venous malformations arise from vessels in the diploic space and are supplied by branches of the external carotid artery, with the middle meningeal and superficial temporal arteries being the main sources [14]. Most of these vascular tumours in the skull contain dilated blood vessels separated by fibrous septa [2, 16, 17]. Usually they are small and asymptomatic, but they can cause pain or present as a palpable deformity [1, 2, 6]. Calvarial venous malformations affecting the roof of the orbit can cause proptosis and even blindness; and those located in the petrous bone may present with deafness or cranial nerve palsies [2, 13–15]. Growth of these neoplasms occurs by expansion of the outer table. Rarely, they can have intracranial extension with few cases reported of erosion of the inner table and dura mater presenting as subdural haemorrhage [16, 17].

**Fig. 6:**
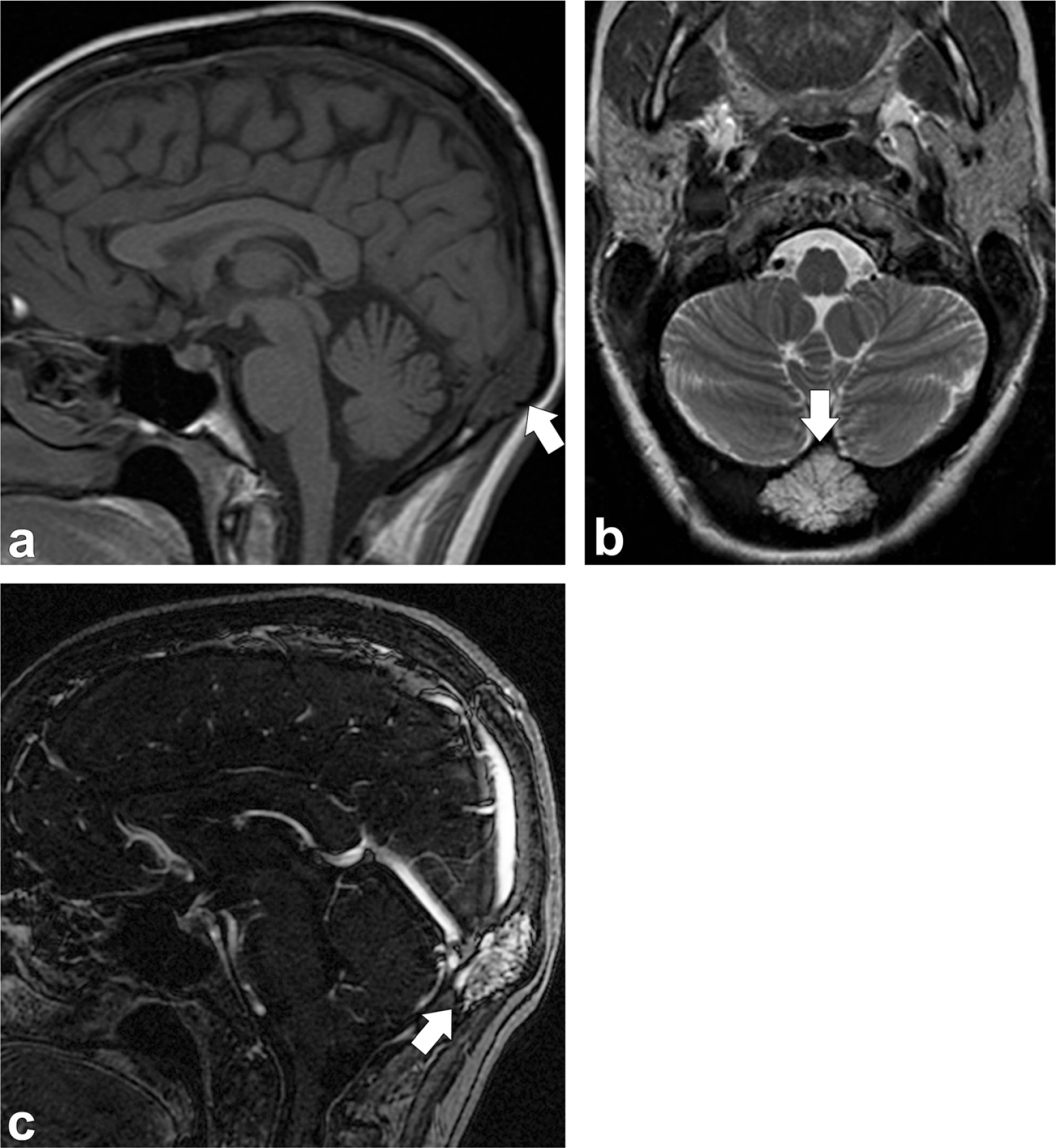
Osseous venous vascular malformation. Sagittal T1-weighted imaging (**a**), axial T2-weighted imaging (**b**) and sagittal contrast-enhanced venogram (**c**) show a hypointense T1, hyperintense T2 and enhancing lesion in the occipital bone (*arrows*)

**Fig. 7:**
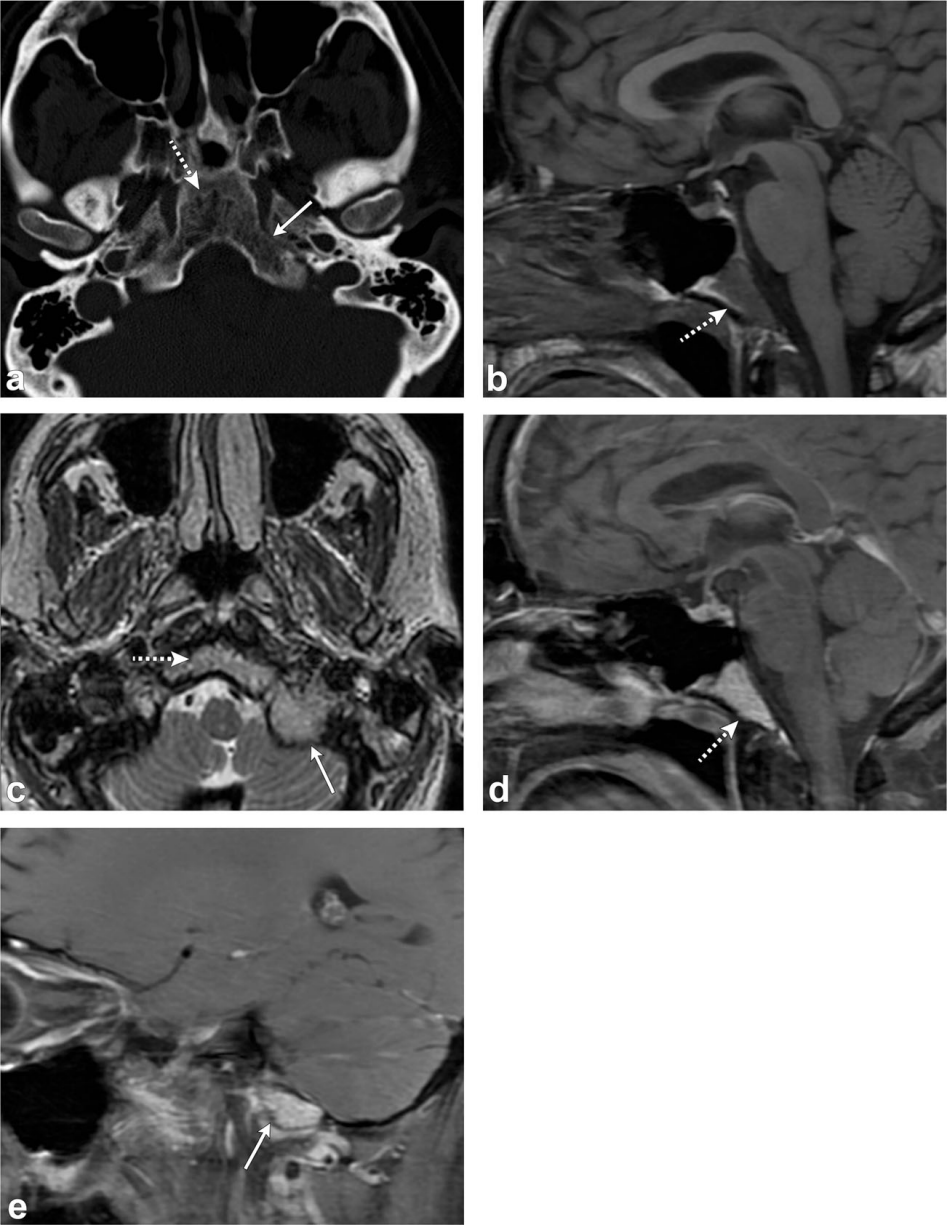
Osseous venous vascular malformation. Axial head CT (**a**) shows osteolytic changes (trabecular appearance) of the clivus (*dashed arrow*) extending into the left occipital condyle (*thin arrow*). Sagittal T1-weighted (**b**), axial T2-weighted (**c**) and post-contrast sagittal T1-weighted images (**d**, **e**) show abnormal signal in the clivus (*dashed arrows*) and left occipital condyle (*thin arrow*) with enhancement

On plain radiographs, this vascular tumour presents as an osteolytic lesion with the characteristic “sunburst” or “honeycomb” trabecular pattern, which is due to thickened trabeculae adjacent to the angiomatous channels [1, 2, 4, 16–18]. CT shows a well-circumscribed intradiploic osteolytic lesion with mild expansion of the outer table and relative sparing of the inner table. A sunburst pattern of trabecular thickening radiating from a common centre is the classic finding [2, 4, 16–18]. On MRI, the serpentine vascular channels can be identified and the appearance depends on the fat content and vascularity of the lesions. They are characteristically isointense to hyperintense on T1-weighted images and hyperintense on T2-weighted images with a “bunch of grapes” appearance (Figs. 6 and 7). Low signal on T1 is due to decreased marrow fat or greater vascular component and has been associated with a more aggressive behaviour. Punctate or reticular low T2 signal may be present representing fibrous tissue or foci of calcification. These bony lesions enhance diffusely after contrast administration. Sometimes they can have an aggressive pattern with a soft-tissue component and may simulate a malignant neoplasm [1, 2, 6, 16–18].

Treatment of these lesions is mainly surgical; embolisation may be performed before surgical intervention to reduce blood loss [2].

### Intraosseous meningioma

**Fig. 8:**
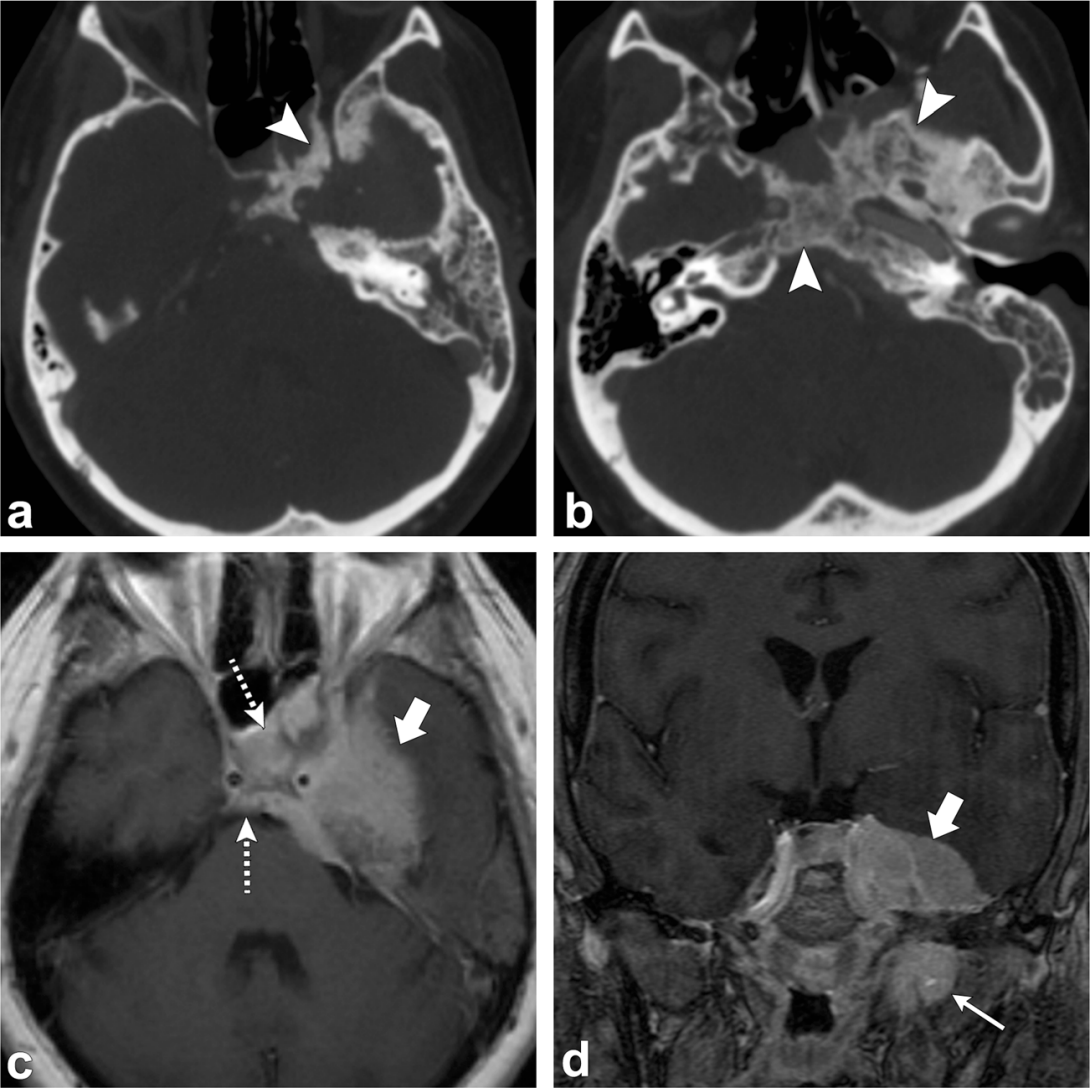
Intradural meningioma. Axial head CT images (**a**, **b**) show hyperostosis of the left sphenoid bone, clivus and petrous portion of the left temporal bone (*arrowheads*). Axial (**c**) and coronal (**d**) post-contrast T1-weighted images show a large enhancing lesion along the left sphenoid wing (*thick arrows*) extending along the clivus and sella (*dashed arrows*), as well as left middle cranial fossa (*short, thick arrow*); there is also extension outside of the calvarium (*thin arrow*)

Meningiomas are the most common extra-axial dural based tumours in middle-aged and elderly patients [1, 2, 6, 19]. The characteristic finding of an intradural meningioma is reactive hyperostosis of the adjacent calvarium (Fig. 8). Occasionally, they can produce marked bone thickening with outward growth of the outer table of the skull [1, 4].

The meningiomas arising outside the dural compartment have been called ectopic, extradural or intraosseous meningiomas. Lang et al. [19] has proposed the term “primary extradural meningioma” for such lesions; this is to differentiate them from primary intradural meningiomas which may have secondary extracranial extension and/or may have metastasised into the skull and adjacent soft tissues.

Intraosseous meningioma is a rare subtype of meningioma that account for <2% of all meningiomas [19–22]. The frontoparietal and orbital regions are the most common locations [2, 19, 20]. Although primary intradural meningiomas occur twice as frequently in women compared to men and predominantly in older adults, intraosseous meningiomas occur with the same frequency in men and women and have a peak in the second decade of life [19]. It is thought that they are due to trapping of embryonic arachnoid cap cells in the developing calvaria in the cranial sutures (particularly the coronal suture) or due to trapping of arachnoid cells in a prior skull fracture or surgical osteotomy [2, 19, 20]. Calvarial intraosseous meningiomas are more prone to develop malignant changes (11%) compared with primary intradural meningiomas (2%) [19, 22].

Osteoblastic or mixed osteoblastic-osteolyic meningiomas compose most of intraosseous meningiomas, with the purely lytic form being the least common [1, 2, 4, 19]. Primary extradural meningiomas are classified as extracalvarial (type 1), purely calvarial (type 2) or calvarial with extracalvarial extension (type 3) [19].

**Fig. 9:**
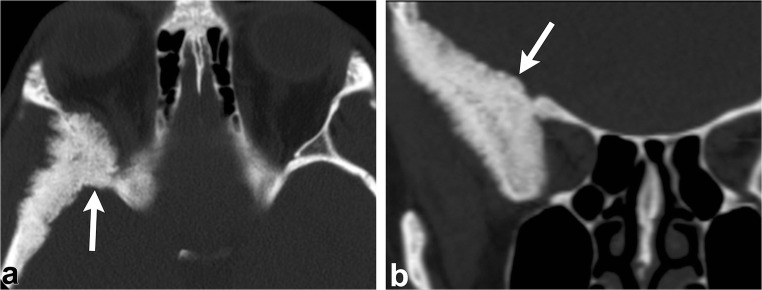
Intraosseous meningioma. Axial (**a**) and coronal (**b**) CT images portray an intraosseous meningioma in the right sphenoid extending into the right lateral orbital wall (*arrows*). Note the expansion of bone and “prickly” border of the lesion

On CT, an intraosseous meningioma typically appears as a sclerotic lesion with associated hyperostosis of the bone, and irregular and spiculated borders (Fig. 9) [2, 4]. On MRI, the tumour has low signal on T1-weighted images and variable signal intensity on T2-weighted images [2, 4, 6]. Meningeal enhancement is rare and is due to adjacent dural irritation or invasion [1, 2, 4]. Intraosseous meningiomas can rarely be osteolytic and in those cases are more commonly malignant [2, 4, 19].

In a symptomatic primary intraosseous meningioma, total tumour removal with a wide surgical resection followed by cranial reconstruction is the treatment of choice. If only subtotal resection is possible due to involvement of critical structures within the orbit, paranasal sinuses, or skull base, the residual tumour should be followed radiologically. Adjuvant radiation therapy is recommended if the residual tumour is symptomatic or if there is evidence of disease progression [2, 19].

### Paget disease

Paget disease (also known as osteitis deformans) is a chronic skeletal disorder characterised by abnormal and excessive bone remodelling. Paget disease is estimated to affect approximately 3–4% of individuals older than 40 years [2, 5, 23–26]. It is a progressive disorder that evolves through various stages or phases of activity. Three major phases are recognised: the lytic phase (incipient active), in which osteoclastic resorption predominates; the mixed phase (active), in which there is both osteoblastic and osteoclastic hyperplasia with predominantly osteoblastic activity; the sclerotic or blastic phase (late inactive), in which the osteoblastic activity gradually declines. This anarchic bone behaviour produces disorganised new bone in a mosaic pattern [2, 6, 23, 24]. Paget affects the skull in 28–42% of cases, particularly the frontal and occipital bones. Other locations affected by Paget are the lumbar spine (30–75%), pelvis (30–75% of cases), sacrum (30–60%) and femur (25–65%). Polyostotic disease is more common than monostotic disease [23, 24].

Skull involvement in Paget disease can lead to increase in head size, hearing loss and entrapment of the cranial nerves in their respective foramina. When the skull base is involved, there is platybasia and basilar invagination in which the tip of the odontoid projects more than 5 mm above the Chamberlain line between the hard palate and basiocciput [23, 24].

**Fig. 10:**
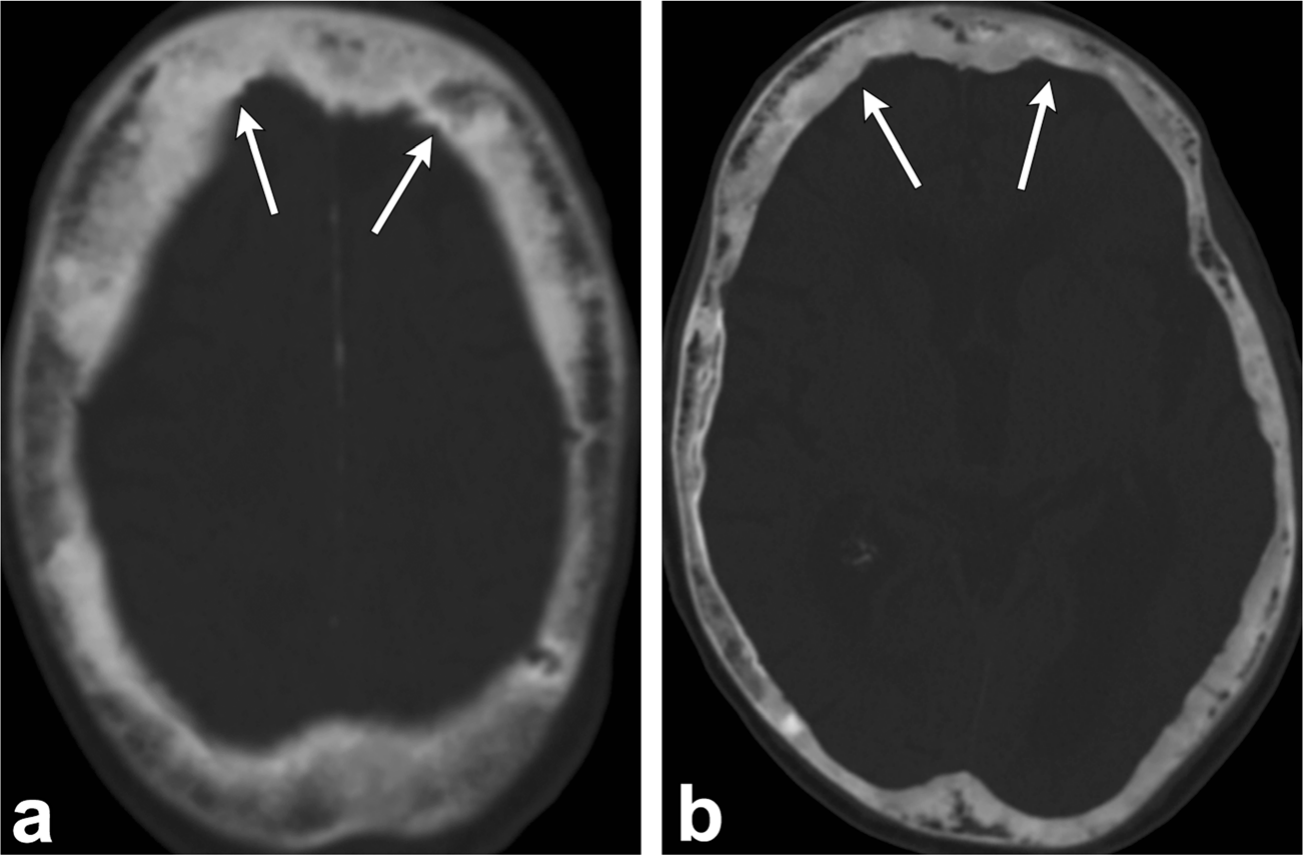
Paget disease. Axial head CT (**a**, **b**) images demonstrate increased cortical density and trabecular thickening (*arrows*)

Early in the disease, osteolytic activity predominates. In this phase, advancing osteolysis presents as large areas of radiolucency in the frontal and occipital bones, known as “osteoporosis circumscripta”. The mixed phase is notable for four cardinal features, which comprise advanced osteolysis, coarsening and thickening of bone trabecula, cortical thickening and osseous widening. In this phase, there is homogeneous enlargement of the skull vault, thickening of the tables and trabecula. The fluffy cotton-wool appearance of the skull is characteristic in this phase with diploic dense lesions in previously sclerotic areas (Fig. 10). Diploic widening is also seen in this phase [2, 5, 6, 23, 24].

As suspected, MRI features vary depending on the stage of the disease. Initially, during bone resorption, high signal intensity can be seen on T2-weighted images, as well as strong enhancement. In this stage, on T1-weighted MR images, the marrow has decreased signal intensity, generally similar to that of muscle; however, it typically contains small to extensive foci of intermixed, normal and maintained yellow marrow. This feature is important because it excludes malignant transformation. In the mixed phase, the yellow marrow signal is maintained in all pulse sequences. In the late blastic inactive phase, the marrow space has low signal intensity on both T1- and T2-weighted images representing sclerosis [2, 5, 6, 23, 24].

Sarcomatous degeneration of Paget has been described with varying frequency depending on the extent of disease. In patients with widespread skeletal involvement, sarcomatous degeneration may occur in 5–10% of cases, whereas in patients with less skeletal involvement, neoplasm can occur in less than 1% of cases [23, 27].

Treatment relies mainly on bisphosphonates [2].

### Calvarial sarcoidosis

Sarcoidosis is an inflammatory disorder of unknown cause characterised by the presence of non-caseating granulomas in tissues. Sarcoidosis involves multiple organs, most commonly the lungs, skin and eyes but may be seen in any organ system, including the musculoskeletal system [28]. Skeletal involvement is an uncommon manifestation of sarcoidosis. When it occurs, bone involvement is usually limited to the vertebra and tubular bones of the hand and feet with the classic lacy lytic appearance. Skull involvement in sarcoidosis is very rare with few cases reported [28–31]. Clinically calvarial sarcoidosis may manifest with headaches and skull tenderness [28–30].

**Fig. 11:**
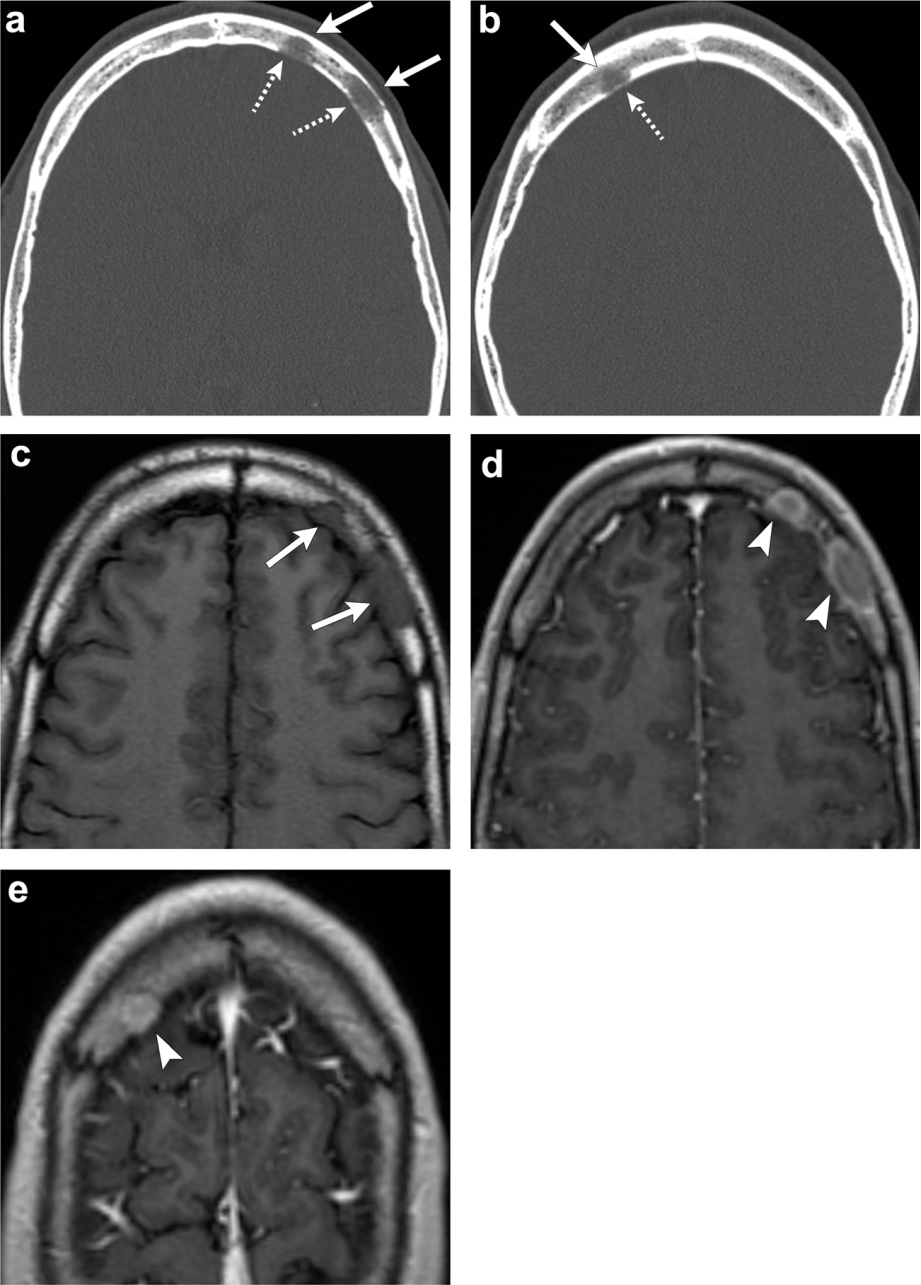
Calvaria sarcoidosis. Axial head CT (**a**, **b**) images show three lytic lesions in the diploic space of the frontal bones (*arrows*) with involvement and thinning of the cortex of the inner tables (*dashed arrows*). Axial T1-weighted imaging (**c**) depicts hypointense lesions (*arrows*). Post-contrast T1-weighted (**d**, **e**) images demonstrate enhancement of these lesions (*arrowheads*)

The lesions are osteolytic with well-demarcated margins and usually multiple. In contrast to metastasis, the calvarial tables are intact. They can also manifest as expansile lesions on CT imaging. MR can be used to assess bone marrow infiltration and associated periosseous soft tissue (Fig. 11). They have variable signal intensity and may enhance after administration of contrast [28–31]. Differential considerations include osseous venous malformation, eosinophilic granuloma, metastasis, as well as chronic infection.

Partial or complete resolution of the sarcoid lesions can occur spontaneously [30]. Treatment is usually indicated when the symptoms include uncontrolled pain, stiffness or bony destruction. Therapy generally consists of oral corticosteroids; methotrexate and hydroxychloroquine can also be used [32].

### Ossifying fibroma

Ossifying fibroma is a benign fibro-osseous tumour made of highly cellular fibro-osseous connective tissue. It is more often seen in children and young adults and can affect the calvaria in approximately 12% of cases. It is a benign slow-growing tumour, but a subset of these tumours tends to be infiltrative and locally aggressive [33, 34]. Three forms of ossifying fibromas have now been distinguished: classical ossifying fibroma, psammomatoid juvenile ossifying fibroma and trabecular juvenile ossifying fibroma. The psammomatoid type of juvenile ossifying fibroma is reported more commonly than the trabecular variety, is more aggressive and has a strong tendency to recur [35].

**Fig. 12:**
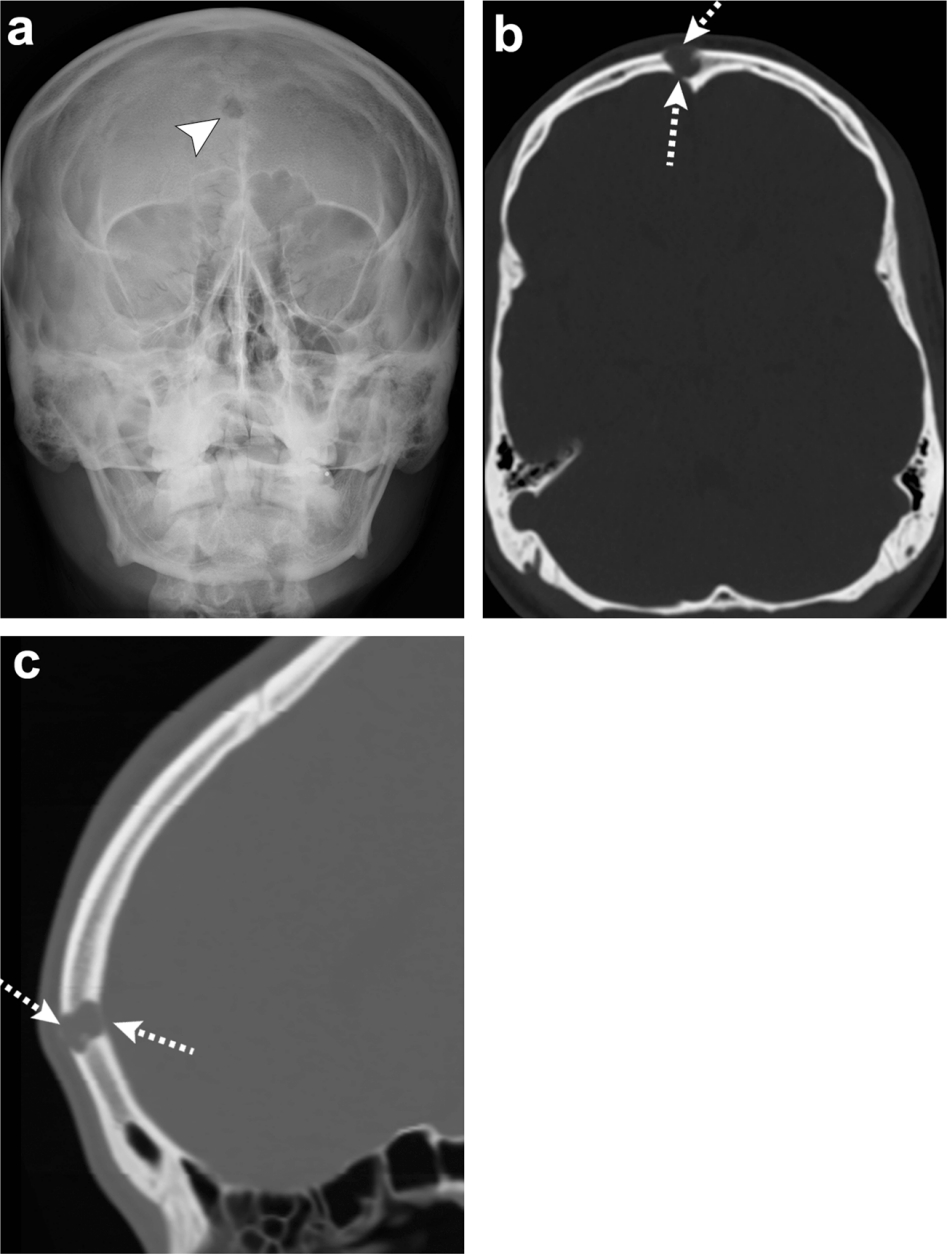
Ossifying fibroma. Water view skull radiograph (**a**), axial (**b**) and sagittal (**c**) head CT images show a lytic lesion in the frontal bone (*arrowhead*) with barely perceptible anterior and posterior cortical margins (*dashed arrows*)

On radiographs, ossifying fibroma appears as a monostotic, round or ovoid, well-demarcated expansile lesion. It may be predominantly cystic or sclerotic depending on the amount of non-calcified versus that of the mineralised regions. On CT, ossifying fibroma is a mass composed of enhancing soft tissue with varying amounts of internal punctate calcifications. Areas of low attenuation caused by cystic changes may be present, and can have internal haemorrhage (Fig. 12) [33–35].

On MRI, the solid component is usually isointense to muscle on T1-weighted images and isointense to hypointense to muscle on T2-weighted images. Post-contrast images show diffuse and heterogeneous enhancement of the solid component and thin peripheral enhancement of the cystic areas [33–35]. Differential considerations include fibrous dysplasia, aneurysmal bone cyst, osteoblastoma and cementum producing lesions such as cemento-osseous dysplasia and cementifying fibromas [34].

Because of its locally aggressive nature and high recurrence rate, early detection and complete surgical removal are essential [34].

### Epidermoid and dermoid cysts

Epidermoid and dermoid cysts are benign slow-growing lesions that may be congenital or acquired from post-surgical or post-traumatic implantation of epidermal or dermal inclusions within the diploe. Clinically, they manifest as non-tender, slowly expanding masses enlarging over years or decades [1, 2, 4–6].

Epidermoid cysts are lined with squamous epithelium and contain remnants of cholesterol and keratin. Intradiploic epidermoid cysts can occur in any part of the skull, but most commonly occur laterally in the parietal and frontal bones. They are usually discovered in patients between 20 and 50 years of age [1, 2, 6].

On CT, epidermoid cysts appear as well-demarcated intradiploic osteolytic lesions with smooth sclerotic margins. These lesions often cause remodelling and expansion of the inner and outer tables. Rarely, they may have increased attenuation on CT, possibly due to haemorrhage, formation of calcium soaps or high protein content (white epidermoids) [1, 2, 4–6, 36].

**Fig. 13:**
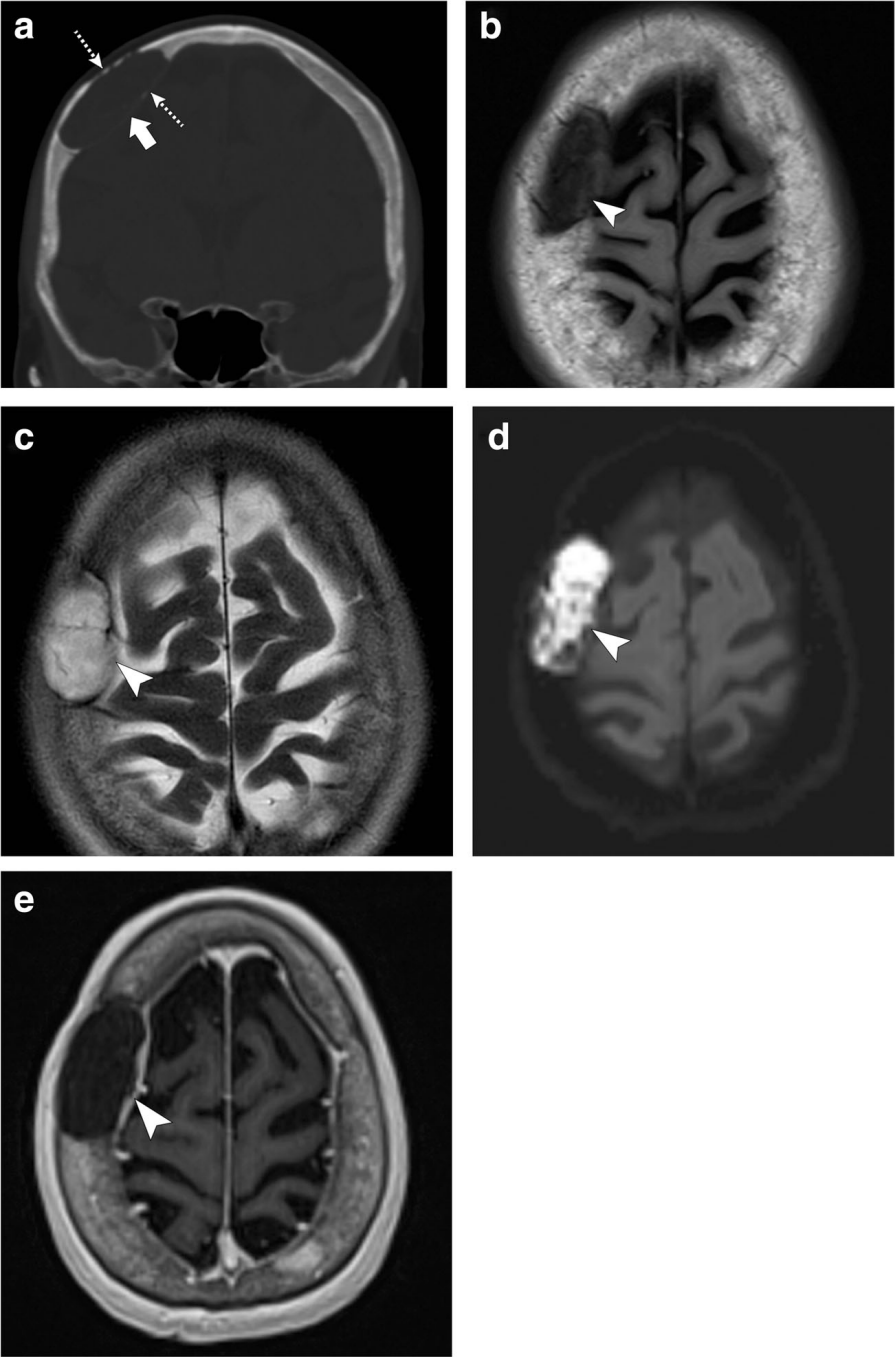
Epidermoid cyst. Axial head CT portrays an expansile lesion in the right frontal bone (*thick arrow*) with thinning of the inner and outer tables (*dashed arrows*). Axial T1-weighted imaging (**b**), axial T2-weighted imaging (**c**), axial DWI (**d**) and axial post contrast T1-weighted imaging (**e**) show that the lesion is hypointense on T1, hyperintense on T2 with restricted diffusion and absent internal enhancement (*arrowheads*). Note that there may be some enhancement along the peripheral margins

On MRI, epidermoids have fluid-signal intensity on T1- and T2-weighted images and have characteristic restricted diffusion on diffusion-weighted imaging (DWI). They do not enhance after contrast administration (Fig. 13) [1, 2, 4–6, 36].

Dermoid cysts are lined by thick squamous epithelium and contain epidermal appendages such as sebaceous glands, sweat glands and hair follicles. They usually present in the first 3 decades of life. These lesions typically involve the midline of the skull near the anterior fontanelle, but may also occur along the posterior and middle cranial fossae [1, 2, 4–6].

On CT, dermoids appear as expansile, osteolytic midline lesions with a soft-tissue component extending into the adjacent soft tissues and intracranially. They have attenuation of lipid material because of their sebaceous secretions. Intralesional calcifications can be present.

**Fig. 14:**
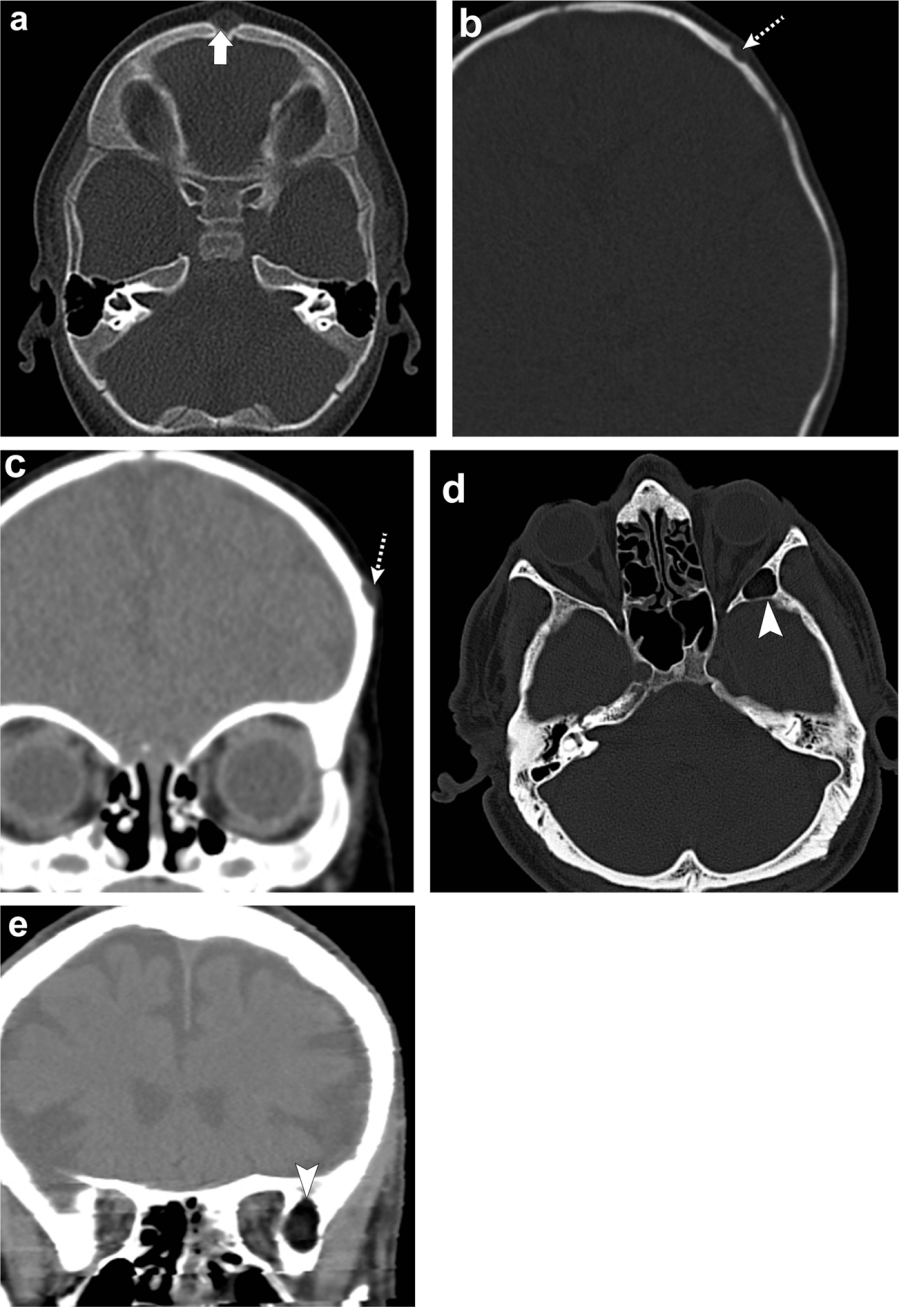
Dermoid cysts. *Patient 1:* Axial head CT (**a**) depicts a midline fat-containing lesion in the frontal region (*thick arrow*). *Patient 2:* Axial bone window (**b**) and coronal soft tissue window head CT (**c**) show a dermoid cyst in the left frontal bone involving the outer table (*dashed arrows*). *Patient 3:* Axial CT (**d**) and coronal head CT portray a fat-containing lesion in the left grater wing of the sphenoid (*arrowheads*)

On MRI, they have a heterogeneous appearance with fatty signal intensity on T1-weighted images, variable signal on T2-weighted images and thick peripheral enhancement after contrast administration (Fig. 14) [1, 2, 4–6, 37].

Treatment of both epidermoid and dermoid cysts is surgical resection; recurrence is uncommon [2].

### Cleidocranial dysostosis

**Fig. 15:**
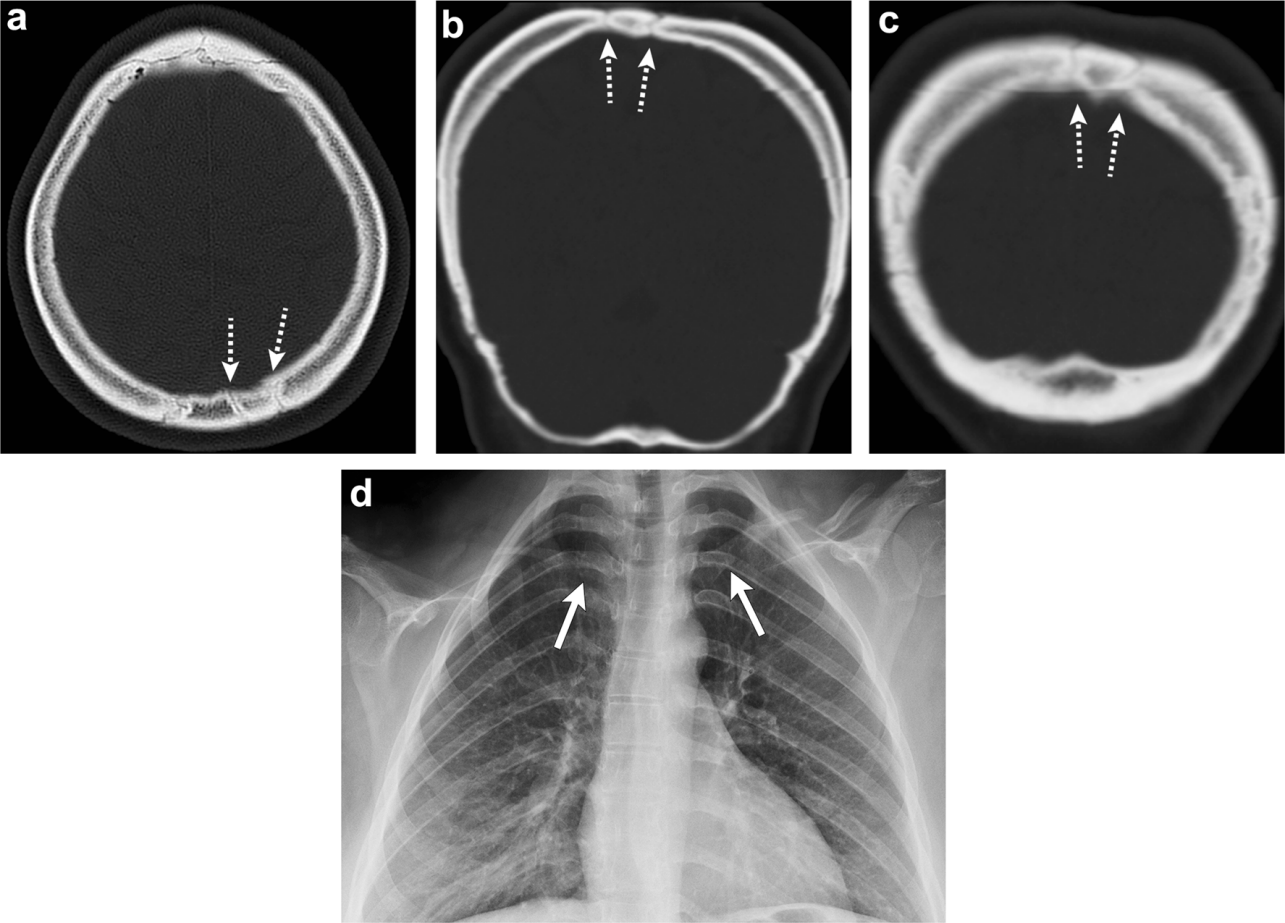
Cleidocranial dysostosis. Axial (**a**) and coronal (**b**, **c**) head CT images show islands of intra-sutural bones (*dashed arrows*) in the lambdoid and sagittal sutures representing wormian bones. Chest radiograph (**d**) in the same patient depicts hypoplasia of the bilateral clavicles (*arrows*), confirming cleidocranial dysostosis

Cleidocranial dysostosis is a rare skeletal dysplasia with autosomal dominant inheritance due to mutation in the *CBAF1* gene. It is a polyostotic disorder characterised by incomplete intramembranous ossification of midline bony structures. It affects the skull, clavicles and eruption of teeth [38, 39]. In the skull, multiple wormian bones due to islands of intrasutural bones in the sagittal and lambdoid sutures are characteristic. Premature fusion of the coronal suture or brachycephaly, as well as frontal and parietal bossing has also been described. Findings outside the skull comprise absent and/or hypoplasia of the lateral clavicle, supernumerary ribs, hemivertebrae, hypoplasia of the iliac bones and short or absent limbs (Fig. 15) [38, 39].

## Malignant calvarial lesions

### Multiple myeloma

Multiple myeloma is a malignant bone marrow disorder characterised by monoclonal proliferation of plasma cells. It is the most common primary skeletal neoplasm in adults above 40 with a higher prevalence in men between the 5th and 8th decades [1, 2, 5, 6, 40].

This disorder causes bone pain with laboratory abnormalities consisting of anaemia, hypercalcaemia, high erythrocyte sedimentation rate with normal C-reactive protein, and high total level of serum protein with a low albumin to globulin ratio due to overproduction of globulins (IgM, IgA). There is also hyperuricaemia due to increased cell turnover and proteinuria with urinary excretion of Bence-Jones light chain immunoglobulin, both of which are nephrotoxic, leading to renal failure [2, 40–42].

Multiple myeloma has four main patterns: disseminated form with multiple round lytic lesions, disseminated form with diffuse osteopenia, solitary plasmacytoma and osteosclerosing fibroma [2, 42]. Multiple myeloma lesions are usually disseminated through the axial skeleton, affecting the vertebrae most commonly, as well as the ribs, skull, pelvic girdle and proximal appendicular skeleton [2, 40–42].

**Fig. 16:**
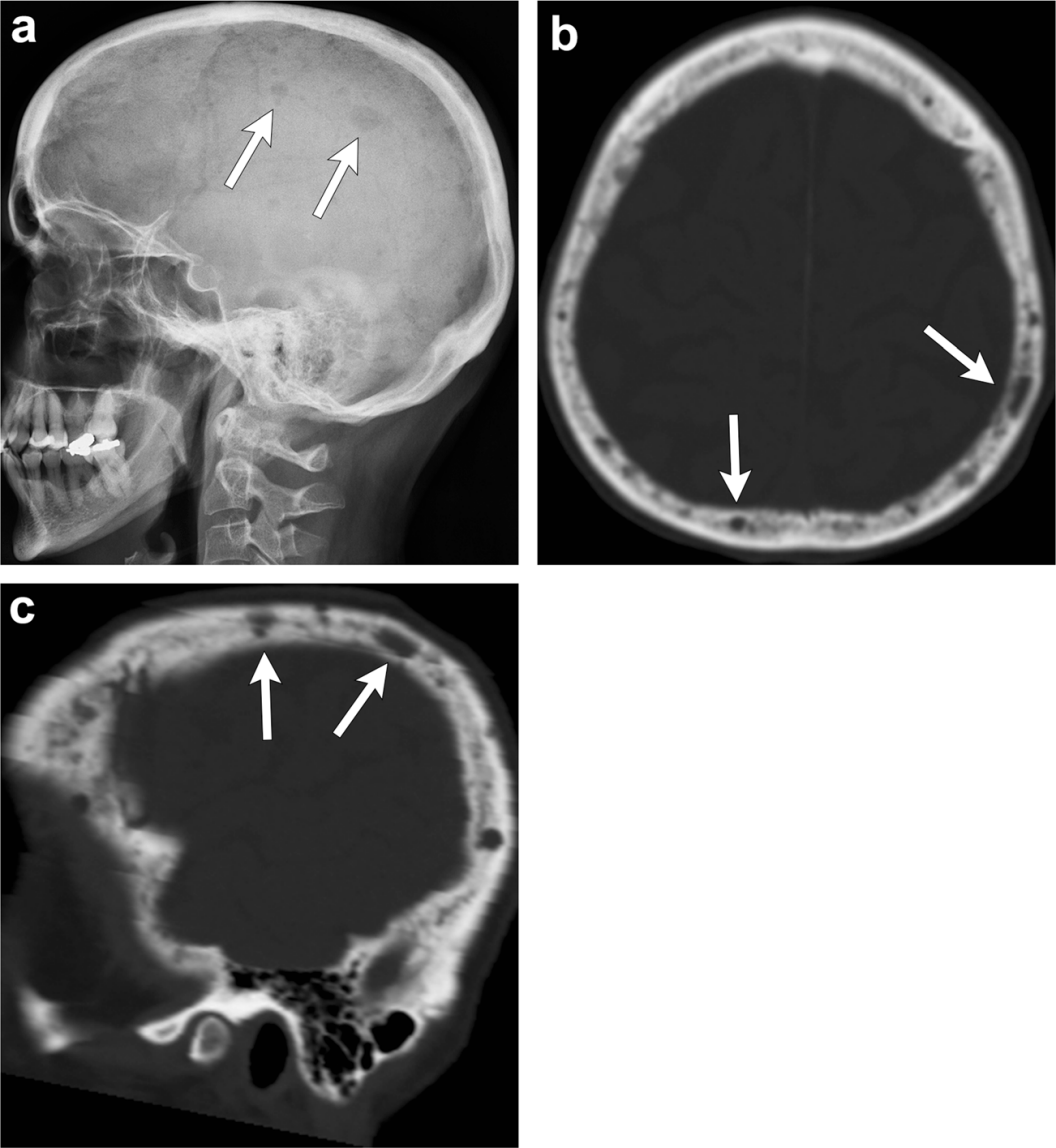
Multiple myeloma. Skull radiograph (**a**), axial (**b**) and sagittal (**c**) head CT show multiple “punched out” lytic lesions in the calvarium (*arrows*), “salt and pepper” appearance

In the skull, radiographs show “punched-out” osteolytic lesions with sharp non-sclerotic margins and endosteal scalloping when lesions abut the cortex (Fig. 16). These lesions may coalesce into larger osteolytic segments. The disseminated form with diffuse osteopenia is less frequently encountered in the skull. Similarly, CT will show multiple lytic foci without a sclerotic rim [2, 5, 6, 40, 42]. Plasmacytoma, which represents the focal solitary form of this neoplasm, is rare in the skull [2, 40]. MRI is superior to detect bone marrow involvement. The lesions are hypointense on T1-weighted images, hyperintense on T2-weighted images and enhance after contrast administration [2, 6, 40–42]. Five different infiltration patterns have been described on MRI with the “salt and pepper” pattern of inhomogeneous bone marrow infiltration being the most common. The other four patterns include: normal bone marrow despite microscopic cell infiltration, focal involvement, homogeneous diffuse infiltration and combined diffuse and focal infiltration [5, 40].

Treatment depends on the stage of the disease and is based on chemotherapy and grafting of haematopoietic cells [2].

### Osteosarcoma

Osteosarcoma is a malignant mesenchymal neoplasm in which the tumour cells produce osteoid or immature bone. It is the second most common primary skeletal tumour after multiple myeloma [1, 43].

Calvarial osteosarcomas may be primary arising de novo or secondary to malignant transformation of Paget disease and radiation therapy. Primary osteosarcoma occurs in the young population with peaks between the 1st and 2nd decades of life. On the other hand, secondary osteosarcoma affects the elderly population [1, 43, 44].

Unlike osteosarcomas of the extremities, calvarial osteosarcomas occur mainly in the 3rd and 4th decades of life [1].

Multiple histological subtypes of osteosarcoma have been described which comprise the conventional or high-grade osteosarcoma, low-grade osteosarcoma, telangiectatic osteosarcoma, periosteal and parosteal osteosarcoma. Many lesions also contain other elements, such as fibroid or chondroid components [45].

**Fig. 17:**
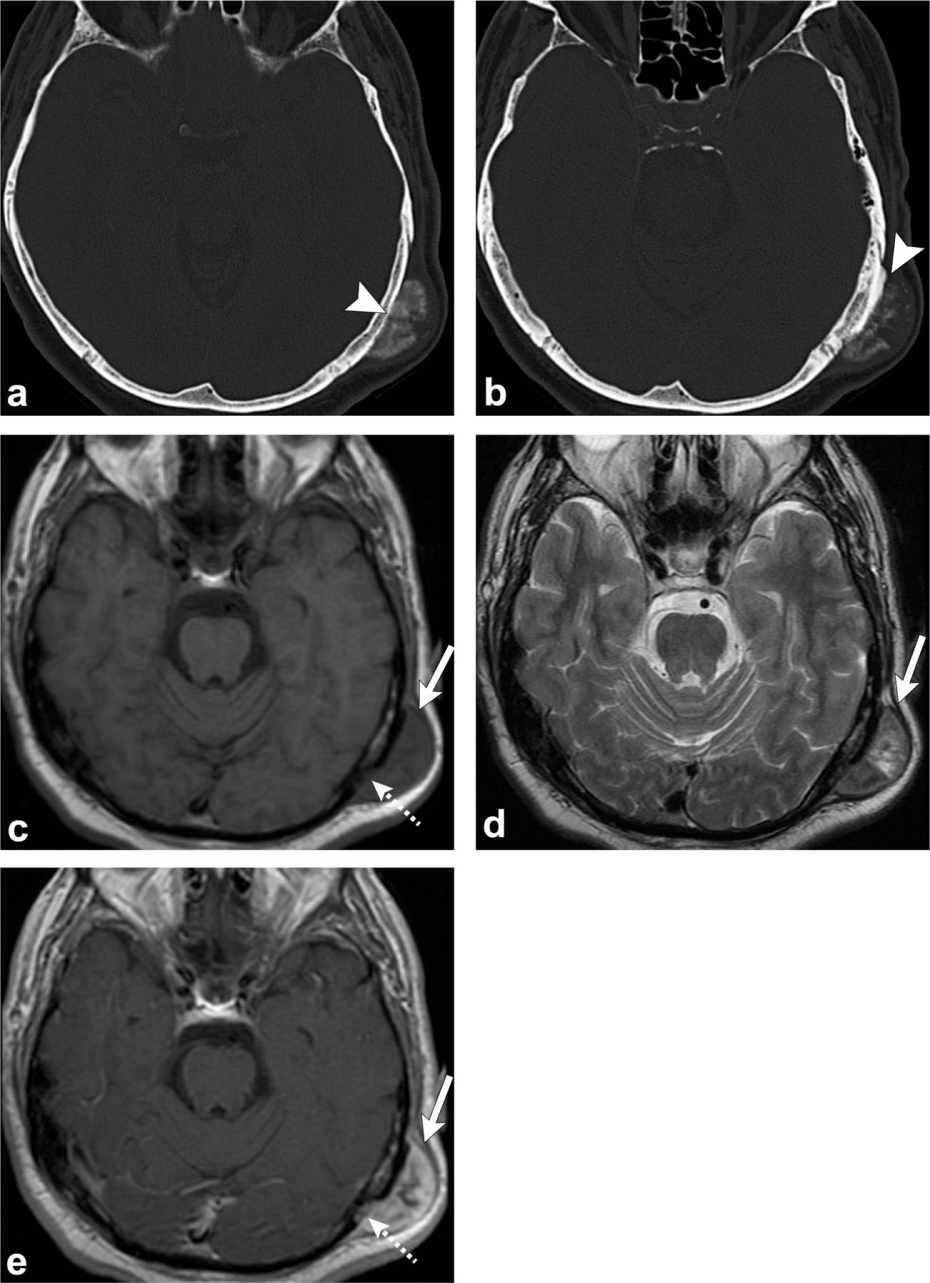
Parosteal osteosarcoma. Axial CT (**a**, **b**) images show a lobulated exophytic mass (*arrowheads*) with central dense ossification in the left temporo-occipital region. Axial T1-weighted (**c**), axial T2-weighted (**d**) and axial post-contrast T1-weighted (**e**) images re-demonstrate a juxta-cortical enhancing lesion abutting the outer table of the left temporo-occipital bones (*arrows*) with a small component extending into the medullary cavity with associated cortical disruption (*dashed arrows*)

The imaging appearance of osteosarcoma is variable depending on the histological type. In the skull, osteosarcoma usually has a lytic appearance with variable amount of osteoid matrix. Also, it has ill-defined borders, aggressive periosteal reaction and an associated soft-tissue mass (Fig. 17) [1, 43, 44].

Approximately 5–10% of osteosarcomas have lung metastasis at presentation and, therefore, imaging of the chest should also be obtained. Treatment requires aggressive surgical therapy followed by chemotherapy [43].

### Metastases

Metastases are the most common malignant bone tumours in adults and are typically seen after the 5th decade of life. Metastases are usually secondary to breast, lung, prostate, kidney and thyroid cancer in adults and to neuroblastoma or sarcomas in children. The most common calvarial metastasis in adults is from breast cancer. In children, the most common calvarial metastases are from neuroblastoma [1, 2, 4–6].

**Fig. 18:**
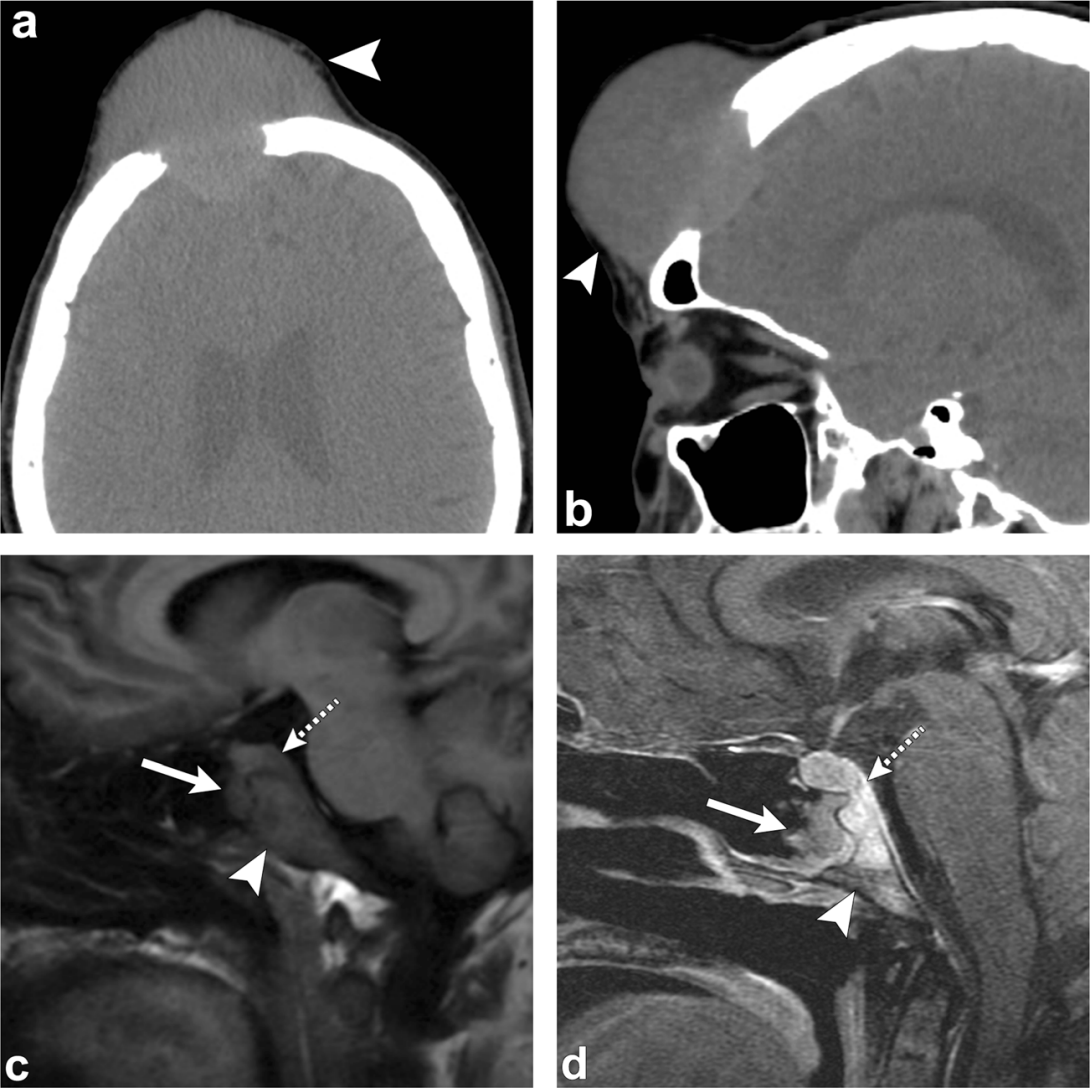
Skull metastasis. *Patient 1:* Axial (**a**) and sagittal (**b**) head CT images show an expansile destructive lesion in the frontal skull (*arrowheads*), pathology proven metastatic renal cell carcinoma. *Patient 2:* Sagittal T1-weighted (**c**) and sagittal post-contrast T1-weighted (**d**) images demonstrate an enhancing lesion in the clivus (*arrowheads*) with soft tissue component extending into the sphenoid sinus (*arrows*) and anterior to the midbrain (*dashed arrow*). This lesion was proven to be metastatic from breast primary

Metastases may have an osteolytic, sclerotic or mixed pattern depending on the primary tumour. Metastases usually present as multiple osteolytic lesions with a soft-tissue component extending into adjacent tissues [4–6]. When single and expanded osteolytic lesions are encountered, metastasis from thyroid or renal neoplasm should be suspected. Sclerotic metastases are seen in prostate cancer. Metastatic lesions are usually hypointense to isointense on T1-weighted images and enhance after contrast administration. The pattern of enhancement is variable and may be homogeneous, heterogeneous, peripheral ring or may lack enhancement in the case of sclerotic lesions. Also, MRI is useful to assess dural invasion and intracranial extension, in addition to disease involving the brain parenchyma (Fig. 18) [1, 2, 5, 6, 46].

Treatment depends on the histological type of the neoplasm. Patients with signs of dura infiltration and related neurological deficit should be offered neurosurgical therapy. Radiation therapy is an alternative in cases of multiple or highly vascularised lesions [2, 47].

### Chordoma

Chordoma is a malignant, locally aggressive tumour that originates from remnants of the notochord. It is seen in the adult population between the 3rd and 7th decades of life [48].

Chordomas are divided into conventional, chondroid and dedifferentiated types. Conventional chordomas are the most common. They are characterised by the absence of cartilaginous or additional mesenchymal components. Chondroid chordomas contain both chordomatous and chondromatous features, and have a predilection for the spheno-occipital region of the skull base. Dedifferentiation or sarcomatous transformation occurs in 2–8% of chordomas [49].

The sacrococcygeal region is the most common location, followed by the clivus, but can occur anywhere along the primitive notochord, including the cervical, thoracic and lumbar spine [48–50]. Chordoma has been considered of low metastatic potential; however, distant metastasis to lung, bone, soft tissue, lymph node, liver and skin has been reported [49].

Skull base chordomas can present with headaches and diplopia and affect men and women with an equal ratio [48].

Clival chordoma is a midline, lytic, destructive, expansile lesion; intratumoral pieces of destroyed bone may be present. When large, the mass can indent the pons with the characteristic “thumb sign” and may elevate the pituitary gland, and cause symptoms related to compression. It can also invade or displace other structures including the cavernous sinus, jugular foramen and the sphenoid and posterior ethmoid sinuses [48].

**Fig. 19:**
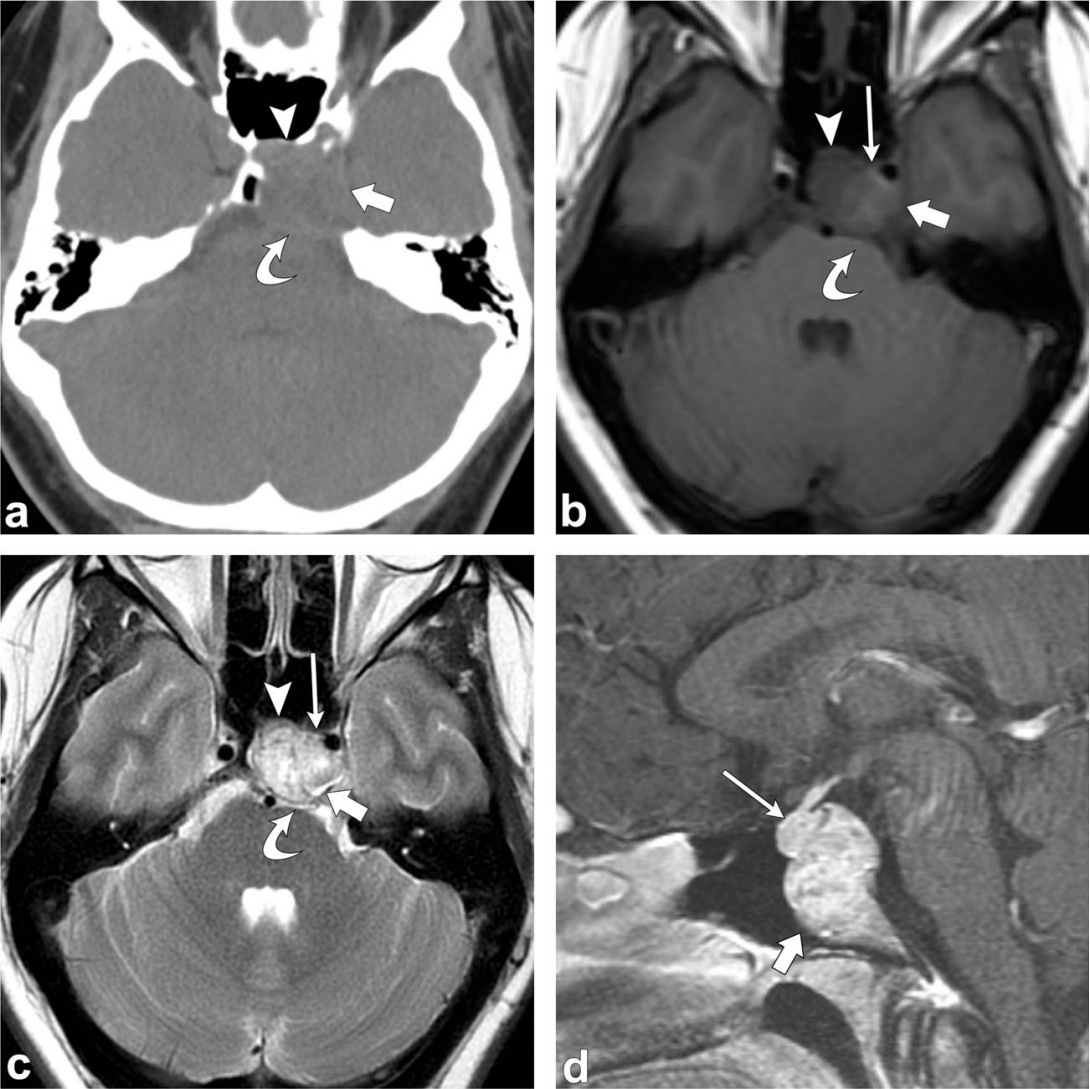
Chordoma. Axial head CT (**a**) shows an osteolytic destructive lesion involving the clivus (*thick arrows*) with extension into the posterior sphenoid sinus (arrowhead) and impression on the pons (*curved arrow*). Axial T1-weighted (**b**), axial T2-weighted (**c**) and sagittal post-contrast T1-weighted (**d**) images demonstrate an expansile mass centred at the clivus extending into the sphenoid sinus (*arrowhead*), left cavernous region (*short, thick arrows*) and partially encasing the left internal carotid artery. This mass enhances and displaces the pituitary gland (*thin, long arrow*) and has mass effect on the left aspect of the pons (*curved arrow*)

On MRI, chordomas have intermediate to low signal on T1-weighted images. Small foci of T1 hyperintensity can sometimes be visualised in the tumour, a finding that represents intratumoral haemorrhage or a mucus pool. They have lobules of high signal on T2-weighted images with intervening septations that are low in signal. Other foci of low T2 signal may be due to calcifications or areas of old haemorrhage. On post-contrast images, they have heterogeneous, avid enhancement; there may be a honeycomb pattern secondary to intratumoral areas of low signal intensity (Fig. 19) [48].

Treatment includes surgical excision and radiotherapy postoperatively in non-resectable tumours. Chordomas have a high rate of recurrence and, as a result, post-treatment follow-up imaging is necessary [48, 50].

### Chondrosarcoma

Chondrosarcomas are slow growing malignant lesions accounting for 6% of the skull base neoplasms. They arise from remnants of embryonal cartilage, endochondral bone or primitive mesenchymal cells. The typical location of this tumour is off-midline, centred at the petro-occipital fissure/synchondrosis [50–52].

**Fig. 20:**
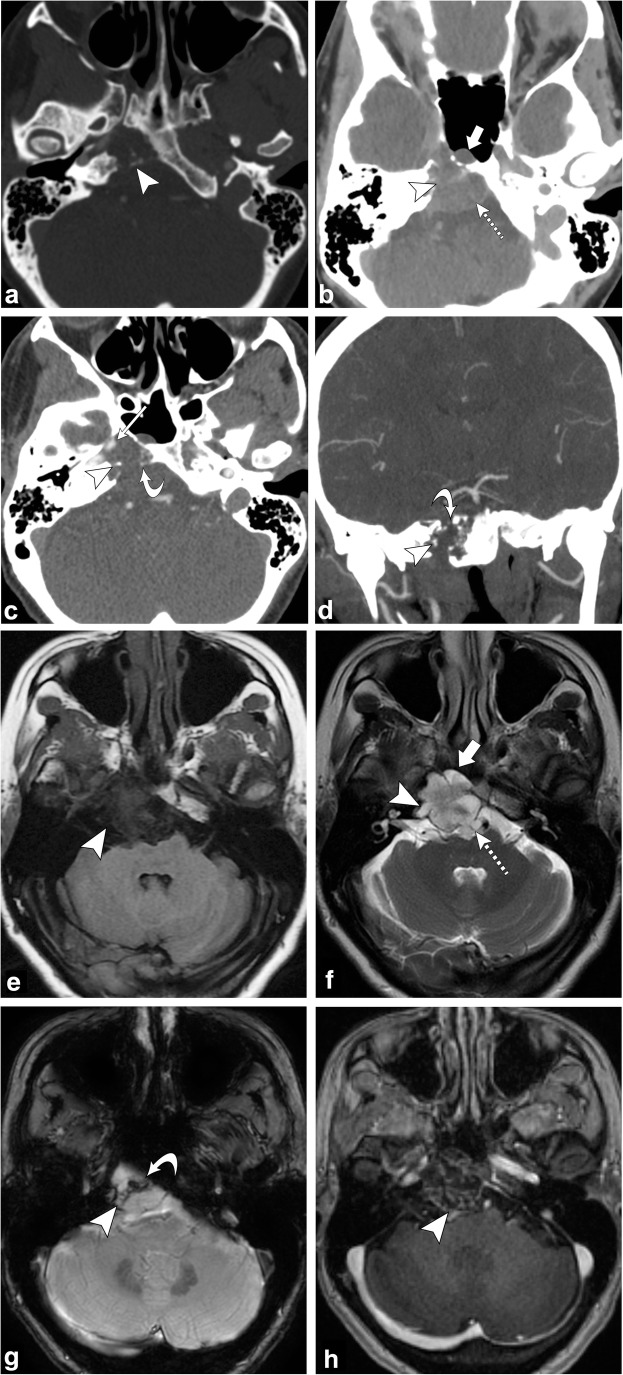
Chondrosarcoma. Axial non-contrast CT (**a**) shows an osteolytic destructive lesion involving the right petro-occipital junction and clivus (*arrowhead*). Axial contrast-enhanced CT (**b**) demonstrates this lesion to have a soft-tissue component extending anteriorly into the sphenoid sinus (*thick arrow*) and posteriorly into the prepontine cistern with mass effect on the pons (*dashed arrow*). Axial (**c**) and coronal (**d**) CT angiogram images demonstrate involvement of the right petrous carotid canal (*arrowheads*) with the lesion surrounding the right internal carotid artery (*thin arrow*). Note the punctate calcifications (chondroid matrix) within the mass (*curved arrow*). Axial T1-weighted (**e**) and axial T2-weighted (**f**) images show a lobulated lesion involving the right petro-occipital junction and clivus (*arrowhead*). Note the extension of the lesion anteriorly into the sphenoid sinus (*thick arrow*) and posteriorly into the prepontine cistern with mass effect on the pons (*dashed arrow*). Axial SWAN (**g**) shows internal foci of low signal, consistent with calcifications (*curved arrow*). Post-contrast axial T1-weighted imaging (**h**) demonstrates heterogeneous (“whorls” of) enhancement (*arrowhead*)

On CT, an osteolytic soft tissue mass is seen at the petro-occipital fissure with the characteristic chondroid matrix calcification (“rings and arcs”) and sharp non-sclerotic transition zone. On MRI, this tumour has low to intermediate signal on T1-weighted images; it has high signal on T2-weighted images; there may be interspersed foci of low signal due to chondroid matrix calcification. Post-contrast images show heterogeneous enhancement, classically with whorls of enhancing lines within the tumour matrix (Fig. 20) [50–52].

The prognosis depends on the extent of the tumour at diagnosis and histological pattern. Conventional chondrosarcomas have a slow growth pattern and a good prognosis. The mesenchymal and dedifferentiated subtypes have an aggressive behaviour. High-grade chondrosarcomas metastasises to lungs and bones more frequently [51]. Surgical resection has traditionally been the mainstay of treatment for intracranial chondrosarcoma. This has been combined with adjuvant radiation and chemotherapy to improve recurrence rates and overall survival [51, 52].

## Systemic conditions affecting the skull

The spectrum of systemic conditions that can affect the skull is broad and can be difficult to diagnose since each tend to have diffuse involvement of the skull. Of these, three systemic conditions are often encountered in clinical practice and familiarity with their main features is key to diagnosis.

### Chronic anaemia

**Fig. 21:**
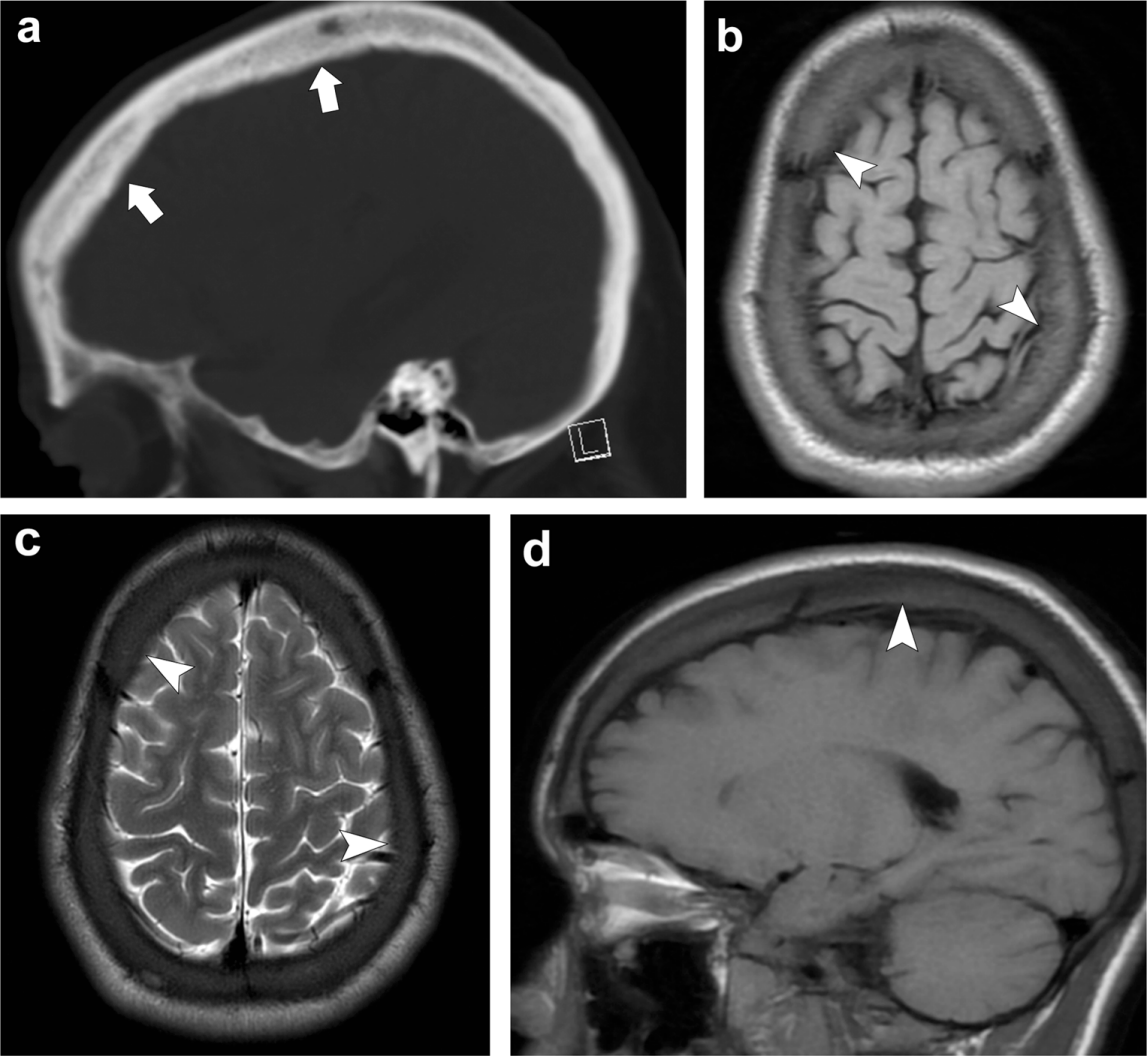
Chronic anaemia. Sagittal head CT (**a**) depicts widening of the diploic space (*thick arrows*). Axial T1-weighted (**b**), axial T2-weighted (**c**) and sagittal T1-weighted (**d**) images portray widening of the diploic space with generalised T1 and T2 marrow hypointensity (*arrowheads*) consistent with red marrow hyperplasia in this patient with sickle-cell anaemia

The skull changes of patients with chronic anaemia are due to red marrow hyperplasia and consist of widening of the diploic space, and thinning and obliteration of the outer table. Before diploic widening, a coarse granular or stippled pattern can be seen in the upper parietal region. When the hyperplastic marrow perforates or destroys the outer table, fine bony spicules perpendicular to the outer table are seen, giving the typical “hair-on-end” appearance. This can be seen with thalassaemia, sickle cell disease and iron-deficiency anaemia (Fig. 21) [5, 53, 54].

### Renal osteodystrophy

Renal osteodystrophy refers to the complex findings seen in the setting of chronic renal insufficiency. Chronic renal failure alters bone metabolism by multiple mechanisms. Phosphate retention and decreased vitamin D conversion leads to hypocalcaemia, which in turn, stimulates the production of parathyroid hormone, causing bone resorption [55–57].

The various skeletal features of renal osteodystrophy have been well documented including generalised demineralisation, thinning of the cortical bone, coarsening of the trabeculae, subperiosteal resorption, bone cysts and pathological fractures [55].

In patients with chronic renal insufficiency, imaging shows diffuse increase in bone density, a finding that is seen more often in the axial skeleton, which has more trabecular bone than cortical bone. It is thought that this diffuse osteosclerosis may be due to the anabolic effect of parathyroid hormone [57].

**Fig. 22:**
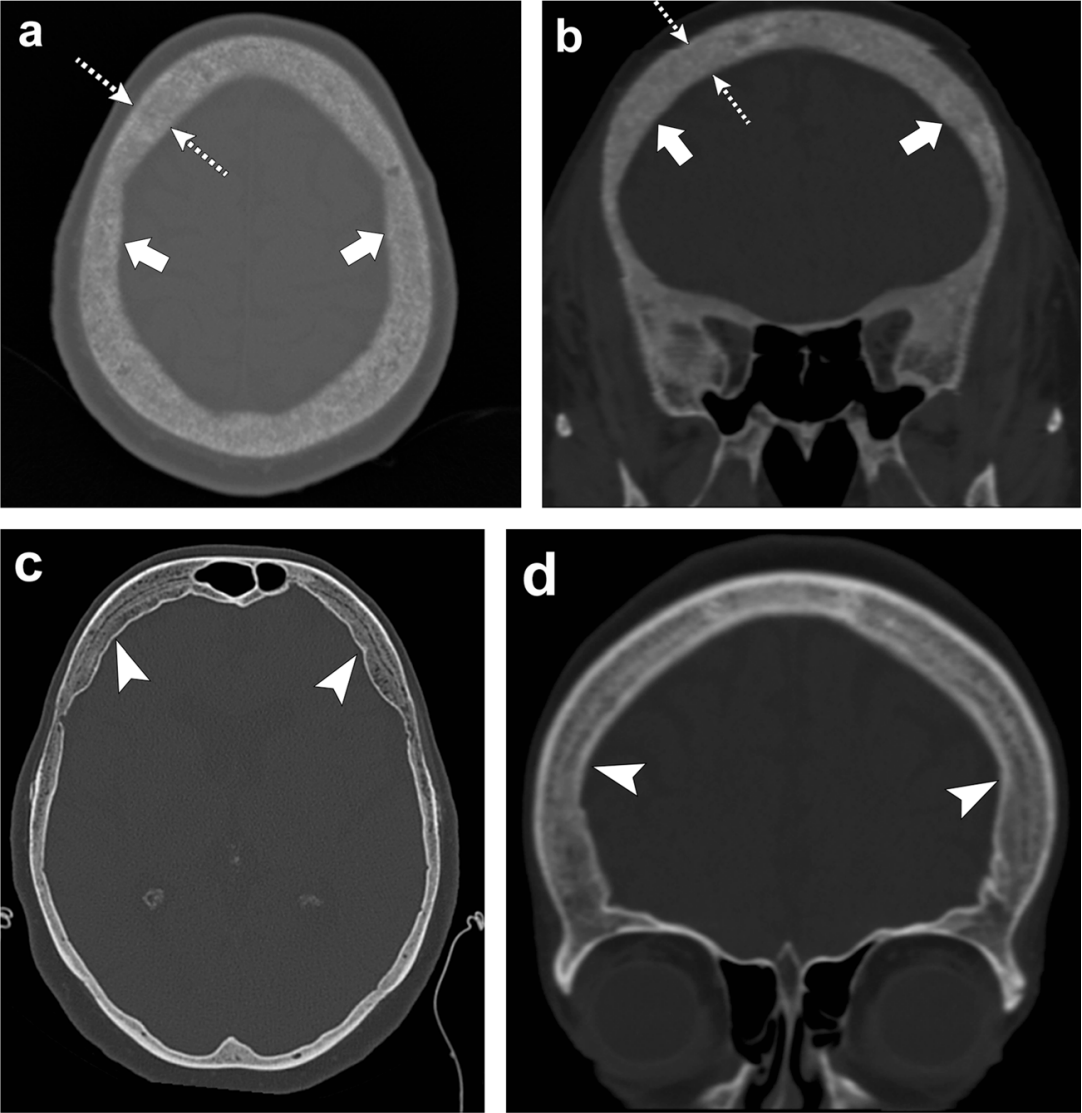
Renal osteodystrophy and osteopenia. *Patient 1:* Axial (**a**) and coronal (**b**) head CT images depict granular de-ossification with a “pepper pot” appearance (*thick arrows*) and loss of distinction of the inner and outer tables (*dashed arrows*) in this patient with renal osteodystrophy. *Patient 2:* Axial (**c**) and coronal (**d**) head CT images show demineralisation of the skull (*arrowheads*) in this patient with osteopenia. Note the relative preservation of the distinction of the inner and outer tables in osteopenia

In the skull, there is diffuse bone thickening with loss of distinction between the inner and outer tables, and granular de-ossification of the skull due to lytic foci interspersed within normal bone, giving the appearance of “salt and pepper” or “pepper pot” skull (Fig. 22) [55–57].

### Osteopenia

Osteopenia is a condition characterised by decreased bone mineralisation. It may arise when bone formation is inadequate or when bone resorption exceeds bone formation. It has multiple causes including congenital, toxic, metabolic and idiopathic. In osteopenia, there is loss of both cortical and trabecular bone, giving rise to a heterogeneous appearance on imaging. In contrast to renal osteodystrophy, the distinction between the inner and outer tables is preserved (Fig. 22) [57].

## Normal variants mimicking pathology (“pseudolesions”)

Skull “pseudolesions” include arachnoid granulations, prominent venous lakes, hyperostosis frontalis, thinning of the parietal bones and surgical burr holes. It is important to recognise these entities, so as to not mistake them for true pathology.

### Arachnoid granulation

Arachnoid granulations, also known as pacchionian granulations are projections of enlarged arachnoid villi into the calvaria or dural venous sinuses. They are involved in the filtration of cerebrospinal fluid (CSF) from the subarachnoid space to the venous system. Histologically, they are composed of dense collagenous connective tissue admixed with clusters of arachnoid cells and a network of delicate vascular space filled with CSF from the contiguous subarachnoid space [5, 58].

The prevalence of arachnoid granulations increases with age. They normally measure a few millimetres, but can grow to fill and dilate the dural sinuses with expansion of the inner table of the skull and are then called “giant arachnoid granulations”. They can occur along any dural venous sinus, but are most commonly along the transverse sinuses, particularly within the middle and lateral portions. The second most common location is along the superior sagittal sinus [5, 58].

**Fig. 23:**
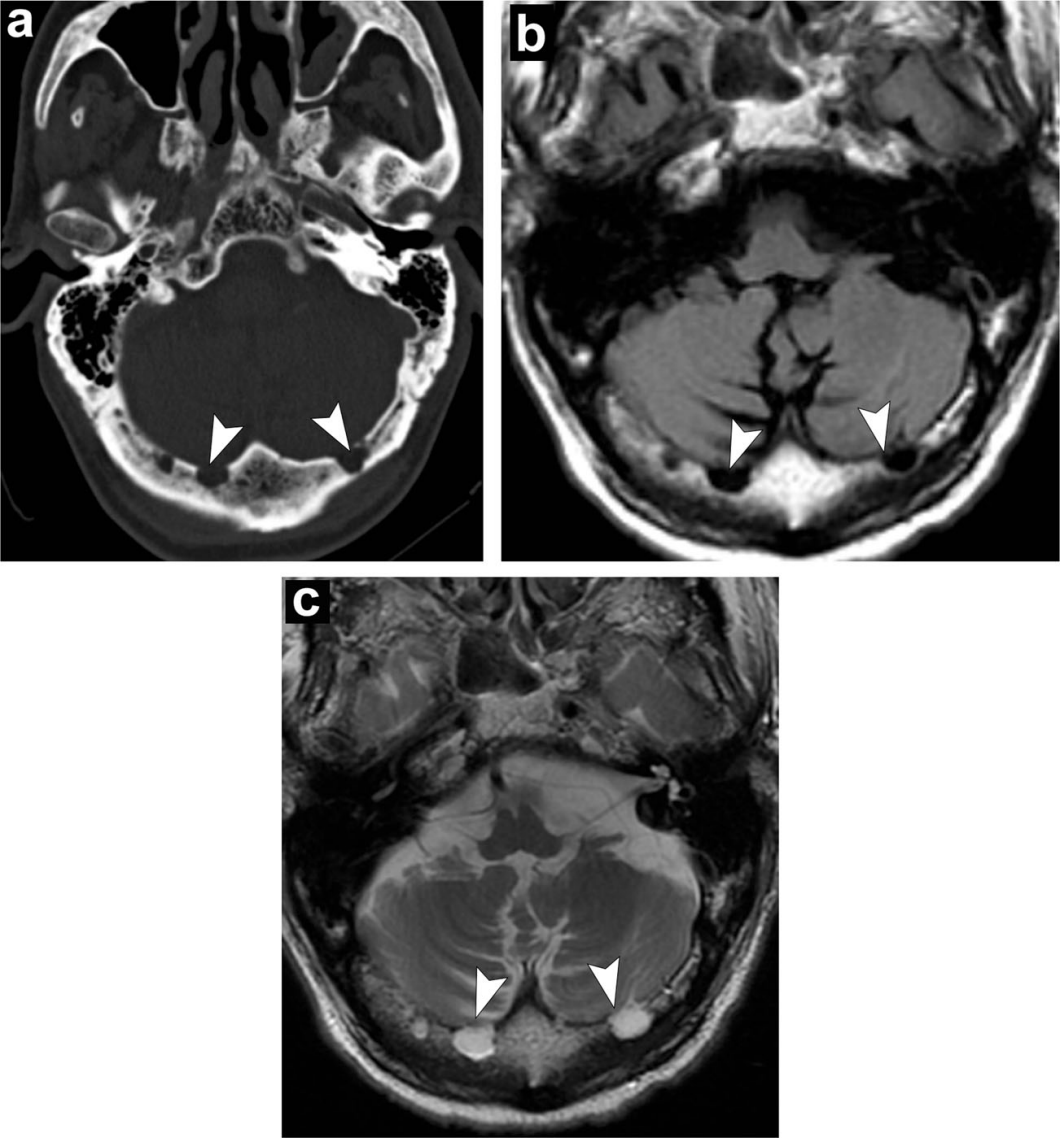
Arachnoid granulations. Axial head CT (**a**) shows well-defined structures along the inner table in the region of the transverse sinus (*arrowheads*). Axial T1-weighted (**b**) and axial T2-weighted (**c**) images demonstrate fluid signal of these structures following CSF along the transverse sinus extending into the occipital bone (*arrowheads*)

On CT, they present as sharply marginated, hypoattenuating structures in close association with a dural venous sinus. On MRI, they generally appear hypointense or isointense to the brain parenchyma on T1-weighted images and hyperintense on T2-weighted images (Fig. 23). They appear as filling defects in the dural sinuses and can mimic a dural venous sinus thrombosis, but are usually easily differentiated given their round well-defined shape and classic location [5, 58].

### Venous channels and venous lakes

Venous channels consist of apertures in the skull through which emissary veins pass, connecting the venous sinuses of the dura mater with veins external to the skull. They look like serpiginous or linear lucencies with sclerotic borders through the skull, and are, therefore, occasionally mistaken for sutures or fractures [59].

**Fig. 24:**
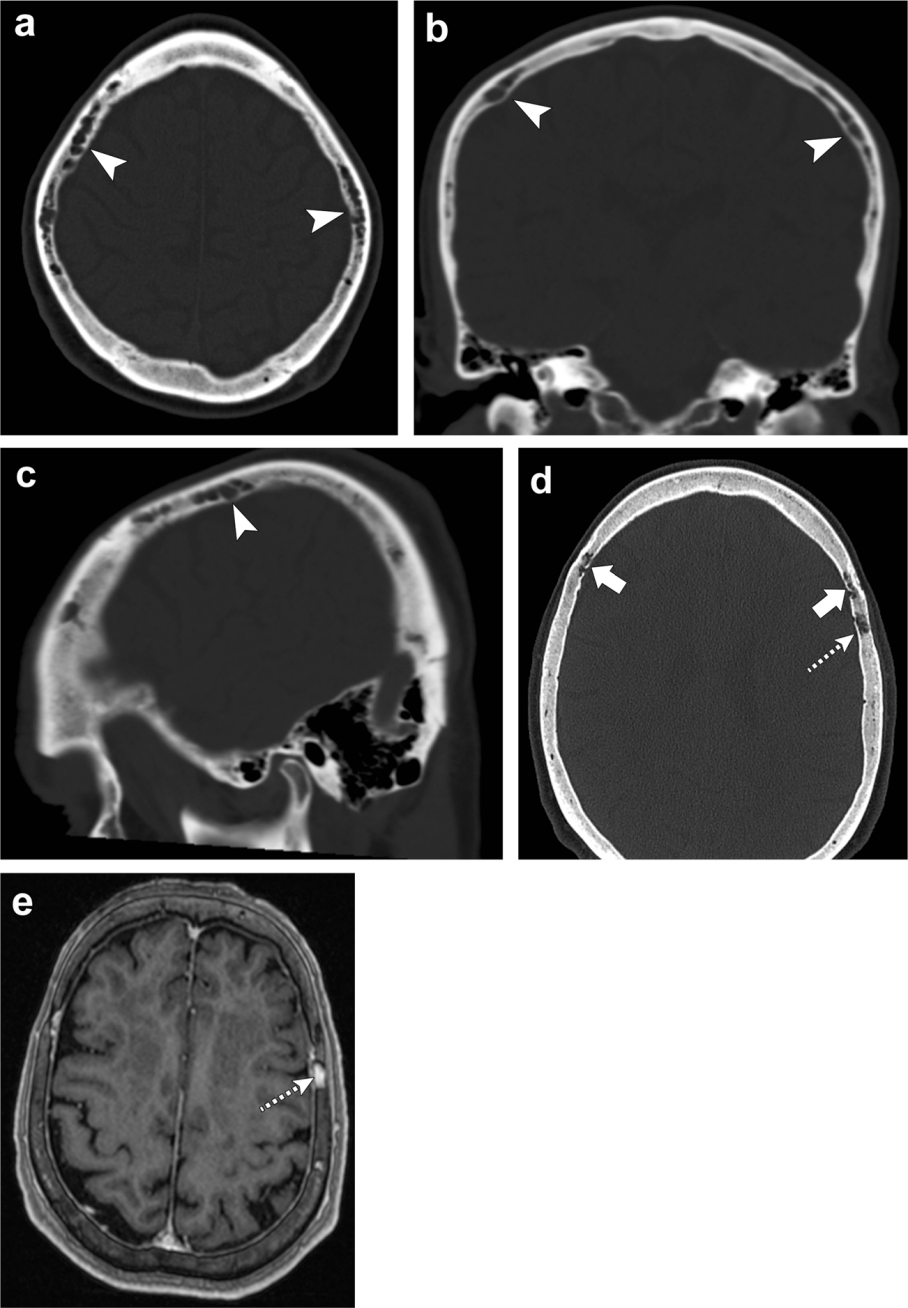
Venous channels and lakes. Axial (**a**), coronal (**b**) and sagittal (**c**) head CT images depict serpiginous structures in the frontal and parietal bones (*arrowheads*) representing prominent venous channels. Axial head CT (**d**) and axial post-contrast T1-weighted (**e**) images show a lucent focus along the left parietal bone with enhancement consistent with a venous lake (*dashed arrows*). There are also mildly prominent serpiginous venous channels in the frontal bones (*thick arrows*)

Enlarged veins within the diploic space are known as venous lakes, corresponding to round or oval lucent foci, frequently along the inner table of the skull (Fig. 24). These normal venous structures show intense enhancement after intravenous contrast administration on both CT and MR [59].

### Hyperostosis frontalis

**Fig. 25:**
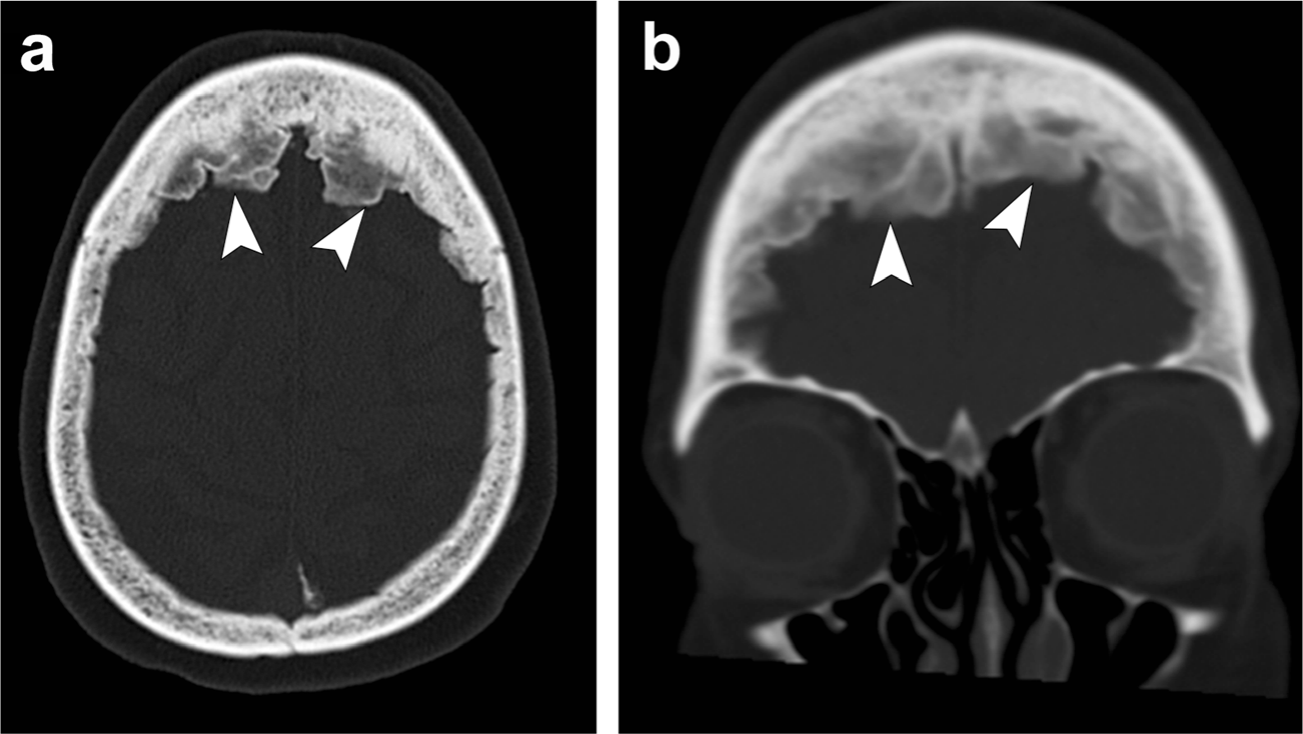
Hyperostosis frontalis. Axial (**a**) and coronal (**b**) head CT images portray irregular thickening of the inner table of the frontal bone consistent with hyperostosis frontalis (*arrowheads*)

Hyperostosis frontalis interna is a benign condition which presents as irregular thickening of the inner table of the frontal bone (Fig. 25). Although the aetiology is not clearly known, oestrogen signalling is thought to be involved and, therefore, most commonly seen in females [5].

### Bilateral parietal thinning

**Fig. 26:**
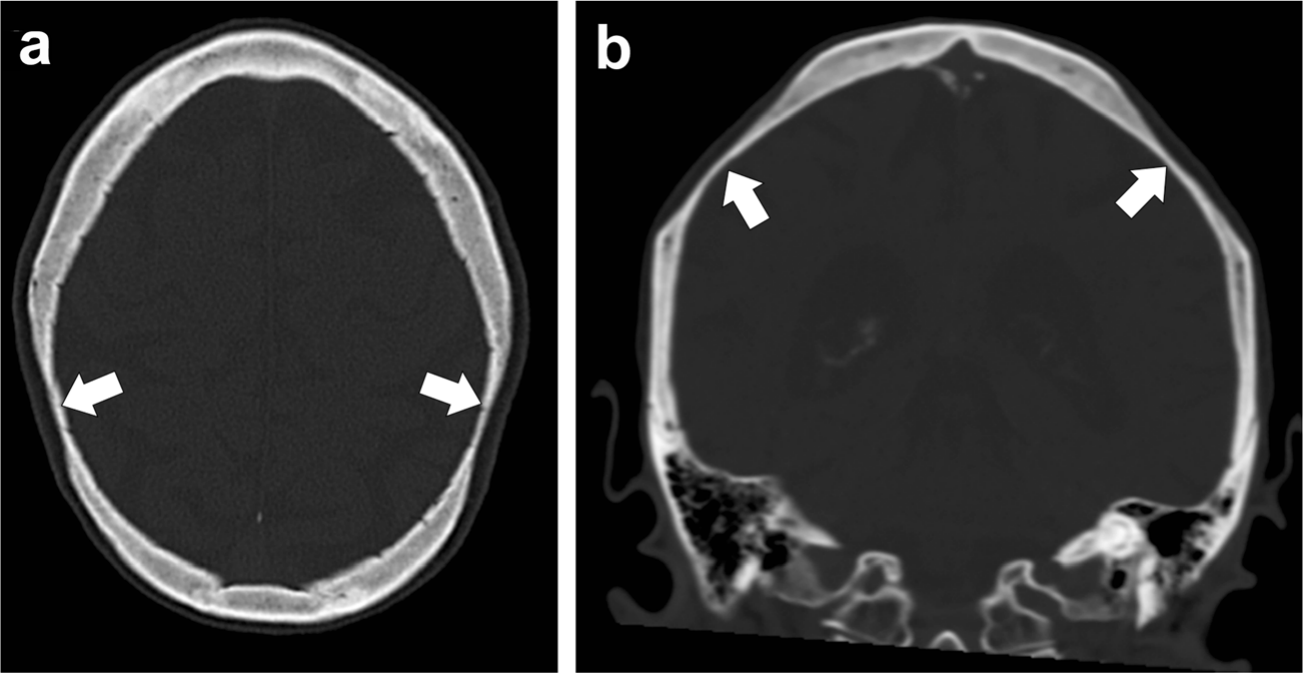
Parietal thinning. Axial (**a**) and coronal (**b**) head CT images shows thinning of the parietal bones (*thick arrows*)

Bilateral parietal thinning is an uncommon condition, with prevalence of 0.25–0.8%. This condition is thought to be due to non-progressive congenital dysplasia of the diploe. Parietal thinning is more common in women than in men. The characteristic site of bilateral parietal thinning is the area between the sagittal suture and parietal prominence. Imaging features in the skull include symmetric thinning of the bilateral parietal bones involving the outer table and diploe, giving a scalloped appearance (Fig. 26). In cases of progressive bone thinning, cranioplasty may be required [60].

### Surgical burr hole

Burr holes appear as well-defined defects in the inner and outer tables of the skull vault on CT or plain radiographs. On contrast-enhanced MRI, the margins of the burr hole usually show enhancement [5].

### Conclusions

As seen, a wide spectrum of benign and malignant neoplasms can involve the calvarium. Benign tumours have well-defined borders with a narrow transition zone and sclerotic margins. On the other hand, malignant tumours have poorly defined margins, a wide transition zone, aggressive periosteal reaction, and lead to dramatic bony destruction with intracranial or extracranial extension. Benign lesions comprise fibrous dysplasia, osteoma, Langerhans cell histiocytosis, venous vascular malformation (formerly known as haemangioma) and Paget disease, as well as several others. Malignant lesions typically affecting the skull are metastases, multiple myeloma, osteosarcoma, chordoma and chondrosarcoma. Systemic diseases may also affect the skull as shown with chronic anaemia, renal osteodystrophy and osteopenia. It is also important to not mistake normal variations of the skull as pathology.

This article has demonstrated that CT and MRI have complementary roles in determining the nature of skull lesions. The recognition of the radiological appearance of calvarial lesions in association with the age, clinical history and symptoms of the patient is essential for the diagnosis, which ultimately impacts management. Some lesions can be left alone with no follow-up, while others need aggressive treatment with resection, radiation and/or chemotherapy.
